# Intelligent Breathing Electronic Skin Inspired by *Nepenthes* for Active Sweat Management, Multimodal Sensing and High-Fidelity Electromyographic Teleoperation Using Machine Learning

**DOI:** 10.1007/s40820-026-02252-2

**Published:** 2026-06-12

**Authors:** Yichen Li, Kai Zheng, Guangtian Zhang, Wentao Chen, Chengtan Liu, Seonho Shin, Bihai Yang, Ran Cai, Bin Hu

**Affiliations:** 1 School of Medical Technology, Beijing Institute of Technology, Beijing, 100081 People’s Republic of China; 2 Key Laboratory of Brain Health Intelligent Evaluation and Intervention, Ministry of Education, Beijing Institute of Technology, Beijing, 100081 People’s Republic of China

**Keywords:** Electronic skin, *Nepenthes*-inspired, Electrospinning, Unidirectional sweat transport, EMG teleoperation

## Abstract

Electronic skin (e-skin), a stretchable, conformable, and multimodal sensing platform, is rapidly advancing in healthcare, robotics, and human–machine interaction (HMI). However, prolonged wear and sweat accumulation at the e-skin-skin interface can introduce signal artifacts, cause discomfort, and eventually lead to interfacial failure. Inspired by the unidirectional liquid transport of the *Nepenthes* peristome, we constructed an intelligent breathing e-skin (SPTL) featuring a Janus bilayer structure that creates a “liquid diode” effect for active sweat transport, achieving a remarkable cumulative one-way transport index of 956.36 while maintaining a dry interface even under profuse perspiration. This device exhibits a high tensile strain of 627%, a breathability of 20.02 mm s^−1^, and a pressure sensitivity of 7.39 kPa^−1^. Furthermore, the SPTL demonstrates versatile multimodal sensing capabilities, including real-time Morse code and non-contact capacitive sensing, while sustaining stable performance over 10,000 pressing cycles (capacitance decay < 3%). As a bio-integrated electrode, SPTL enables high-fidelity acquisition of electroencephalogram (EEG), electromyogram (EMG) and electrocardiogram (ECG) signals, significantly outperforming commercial Ag/AgCl electrodes. Integrated with machine learning algorithms and EMG signals captured by the SPTL, it facilitates precise teleoperation of a quadruped robot and handwritten letter recognition with over 95% accuracy. This bionic strategy provides a versatile solution for intelligent, breathable, and multimodal bio-integrated interfaces.

**Supplementary Information:**

The online version contains supplementary material available at 10.1007/s40820-026-02252-2.

## Highlights

- A *Nepenthes*-inspired Janus e-skin (SPTL) was developed to achieve active unidirectional sweat transport while maintaining high breathability, waterproofness, and biocompatibility for prolonged skin dryness.
- The SPTL offers 627% stretchability and multimodal sensing (including Morse code and non-contact capacitive sensing) and enables high-fidelity acquisition of electroencephalogram, electromyogram, electrocardiogram, and electrooculogram signals.
- By integrating machine learning, SPTL-based sensing allows for precise electromyogram control of a quadruped robot and letter recognition with >95% accuracy.

**Supplementary Information:**

The online version contains supplementary material available at 10.1007/s40820-026-02252-2.

## Graphical abstract

**Figure:**
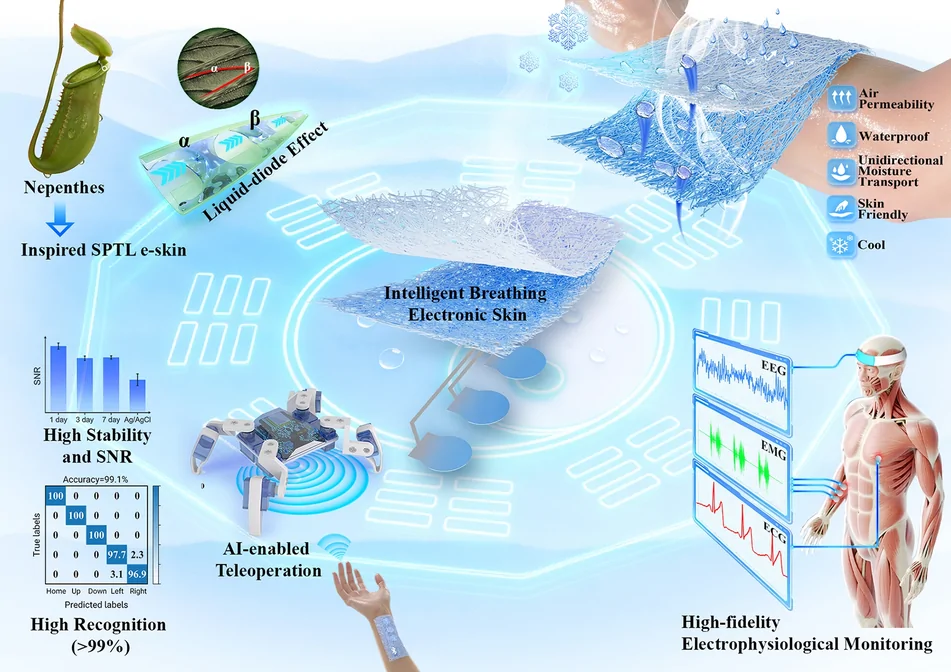

## Introduction

Natural evolution can be viewed as an exquisite encyclopedia of engineering design, providing compelling inspiration for developing intelligent bio-interfaces. The pitcher plant (*Nepenthes*) features a unique peristome surface with hierarchical microstructures, where directionally aligned crescent-shaped scales and step-like grooves facilitate ultrafast, unidirectional liquid transport [1]. This behavior is governed by asymmetric capillary forces and Laplace pressure gradients to lock liquid in place and suppress backflow. Such a structure-guided intelligent liquid-management strategy, rather than one reliant on chemical gradients, can inspire the design of multifunctional interfaces. Electronic skins (e-skins) have become indispensable for daily health monitoring [2–4], clinical diagnostics [5–7], and human–machine interaction (HMI) [8–16]. However, traditional interface configurations often suffer from conductivity degradation, interfacial adhesion failure, and skin irritation caused by sweat accumulation, compromising long-term wearability and signal fidelity. Consequently, there is an urgent need to develop e-skin interfaces that can “breathe intelligently” like *Nepenthes*, actively and directionally wick sweat, and sustain stable performance over prolonged use.

To address the discomfort and signal distortion caused by sweat accumulation during long-term wear, membranes with asymmetric wettability for unidirectional water transport (UWT) have attracted significant attention. Recent research has made significant strides in optimizing these structures for moisture management and functional integration. For instance, Yang et al. balanced environmental sustainability with comfort by creating biodegradable composite fabrics [17], while Yu et al. mimicked bamboo structures to construct nanochannels that significantly enhanced vertical moisture transmission [18]. Regarding multifunctionality, Lei et al. engineered MXene-modified membranes for passive evaporative cooling [19], and Chen et al. utilized pine-needle-like ZnO nanorods to combine moisture wicking with air filtration capabilities [20]. In wearable electronics, Zhang et al. achieved piezoelectric motion monitoring within breathable bilayer fibers [21], and Yang et al. employed bio-inspired microstructures to improve the sweat drainage and interfacial adhesion of hydrogel electrodes [22]. Additionally, Dong et al. [23] and Wang et al. [24] employed superhydrophobic coatings and porous nanofiber networks, respectively, to endow stretchable electrodes with self-cleaning properties and breathability. In fact, recent advances in breathable and stretchable epidermal electronics, particularly textile-based bioelectrodes, have demonstrated exceptional capabilities for continuous health management, exhibiting robust performance even under complex conditions such as heavy sweating and electromagnetic interference [25–28]. Concurrently, the integration of advanced functional materials, such as bio-inspired nanofibers and conductive hydrogels, has driven significant breakthroughs in high-fidelity electromyogram (EMG) monitoring and robust human–machine interfaces [29–32]. However, most UWT strategies remain confined to passive textiles or air filters, lacking deep integration with high-performance flexible circuits necessary for complex HMI. Furthermore, existing breathable devices typically rely on passive evaporation through pores. Under profuse perspiration, this mechanism is insufficient, resulting in sweat accumulation, liquid backflow, and subsequent short circuits or signal artifacts [33, 34]. Meanwhile, hydrogel-based solutions, despite their high signal quality, suffer from dehydration and performance degradation over time [35, 36].

In this work, drawing functional inspiration from the unidirectional water-transport capability of the Nepenthes peristome, we developed an e-skin by integrating a Janus substrate with liquid metal (LM) screen printing. By translating this macroscopic anti-backflow concept into a transmembrane Janus-driven mechanism, the fundamental innovation of this work lies not merely in the standalone application of these materials, but in their unprecedented synergistic integration to resolve the critical bottleneck of sweat-induced signal artifacts during long-term wear. The e-skin (SPTL) features a bilayer architecture comprising a hydrophobic styrene–butadiene–styrene (SBS) inner layer (skin side) and a hydrophilic polyacrylonitrile (PAN)/thermoplastic polyurethane (TPU) outer layer (denoted as PT). This asymmetric wettability gradient generates a liquid-diode effect, spontaneously driving directional sweat transport (one-way transport index up to 956.36) to ensure a dry skin interface while preventing external moisture penetration. Beyond moisture wicking, the device exhibits high-fidelity electrophysiological signal acquisition, a tensile strain of 627%, an air permeability of 20.02 mm s^−1^, and a high sensitivity of 7.39 kPa^−1^. Notably, the device demonstrates exceptional durability (relative capacitance decay < 3% after 10,000 cycles) and rapid response/recovery times (20–30 ms). By integrating machine learning algorithms, we constructed an electromyogram (EMG) teleoperation system that maps human hand kinematics to a quadruped robot. This work provides a versatile strategy for constructing next-generation bio-integrated interfaces, bridging the gap between biological intelligence and robotic execution in extreme scenarios.

## Experimental Section

### Materials

Polyvinylpyrrolidone (PVP, Mw ≈ 1,300,000), ethanol, N,N-dimethylformamide (DMF), and tetrahydrofuran (THF) were purchased from Macklin (China). The liquid metal EGaIn (75 wt% Ga and 25 wt% In) was supplied by Shenyang Jiabei Commercial Technology Co., Ltd. (China). Polyacrylonitrile (PAN) and thermoplastic polyurethane (TPU) were obtained from Qingdao Nuokang Environmental Protection Technology Co., Ltd. (China). Styrene–butadiene–styrene (SBS) was purchased from Hefei Yuanli Instrument Technology Co., Ltd. (China). Release paper was sourced from Tianjin Kemai Biotechnology Co., Ltd. (China). Deionized (DI) water was used throughout the experiments.

### Fabrication of SPTL

**Preparation of LM Ink:** First, 0.5 g of PVP was dissolved in 9.5 g of ethanol and stirred at 70 °C for 6 h to form a clear surfactant solution. Subsequently, 1 mL of this solution was mixed with 3 g of LM. The mixture was sonicated using a probe ultrasonic instrument (1000Y, Shanghai Hanno Instrument Co., Ltd.) at 50% amplitude to obtain the dispersed LM ink.

**Preparation of the SPT Membrane via Electrospinning:** The bilayer substrate (referred to as SPT) was fabricated using a sequential electrospinning process. First, for the hydrophobic layer, 15 wt% SBS was dissolved in a mixed solvent of THF and DMF (3:1, w/w), and stirred at 60 °C for 4 h. The above solution was loaded into a syringe with a 22 G needle (inner diameter 0.41 mm). Electrospinning was conducted with a voltage of 20 kV applied to the needle and -2 kV to the collector. The flow rate was maintained at $1 \mathrm{m}\mathrm{L} {\mathrm{h}}^{-1}$ for 3 h to obtain the SBS nanofiber film.

Then, for the hydrophilic layer, 15 wt% PAN and 2 wt% TPU were dissolved in DMF and stirred at 60 °C for 24 h. This solution was electrospun onto the pre-formed SBS film using a 22 G needle (inner diameter 0.41 mm). The voltage parameters were set to 18 kV (needle) and -2 kV (collector), with a flow rate of $10 \,{\mathrm{m}}{\mathrm{L}}\, {\mathrm{h}}^{-1}$ for 1 h. All parameters were controlled using an electrospinning system (HED-02, Qingdao Nuokang Environmental Protection Technology Co., Ltd.).

**Fig. 1:**
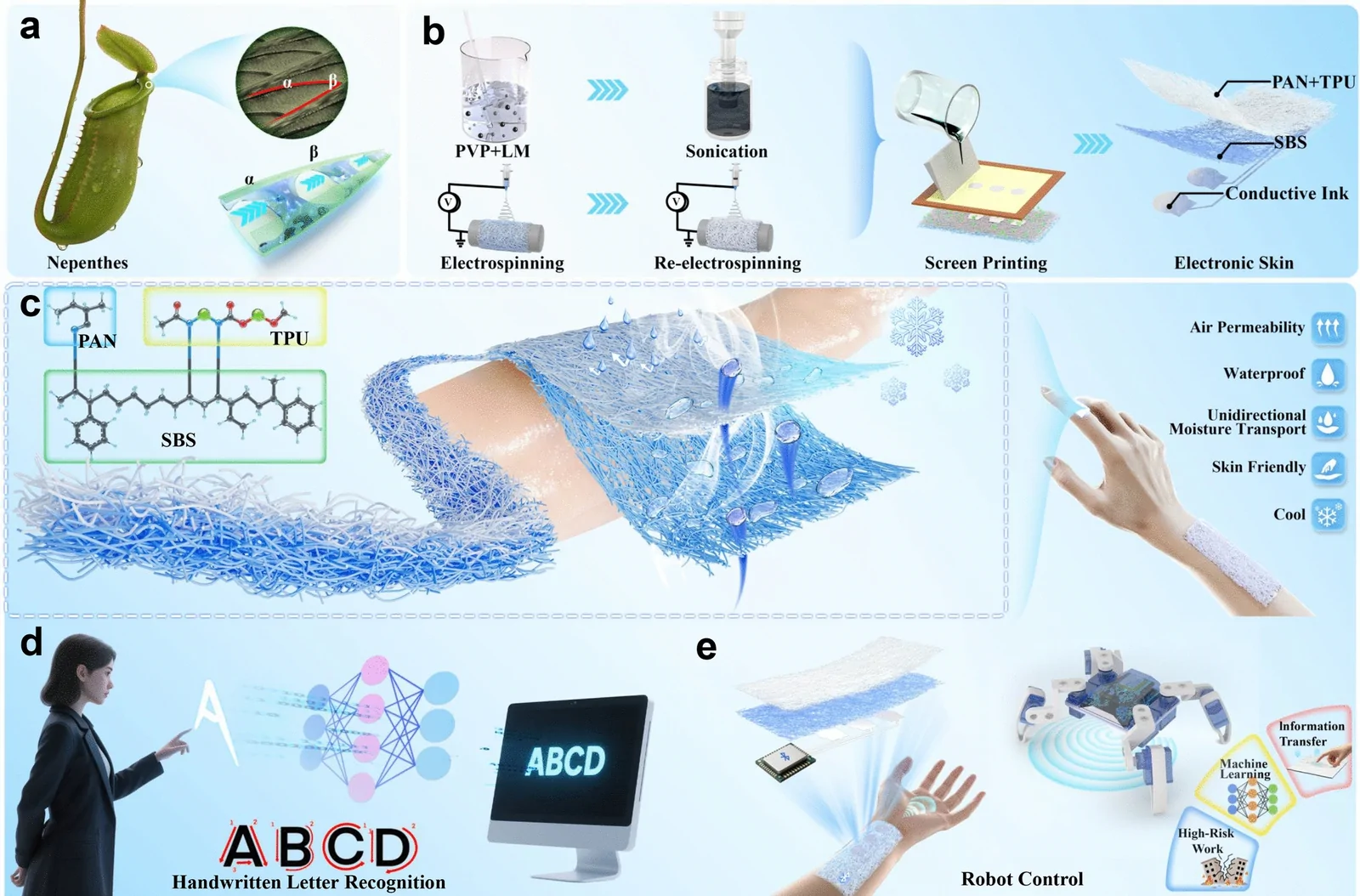
Overview of the bio-inspired design strategy, fabrication process, and applications of the SPTL e-skin. **a** Schematic illustration of the bionic *Nepenthes* structure and its transport mechanism. **b** Schematic of the SPTL fabrication flow. **c** Performance characteristics of the SPT membrane. **d** Schematic of the handwritten letter recognition pipeline based on SPTL-4. **e** Schematic illustrating EMG teleoperation of the quadruped robot using the SPTL-4 e-skin

**Fig. 2:**
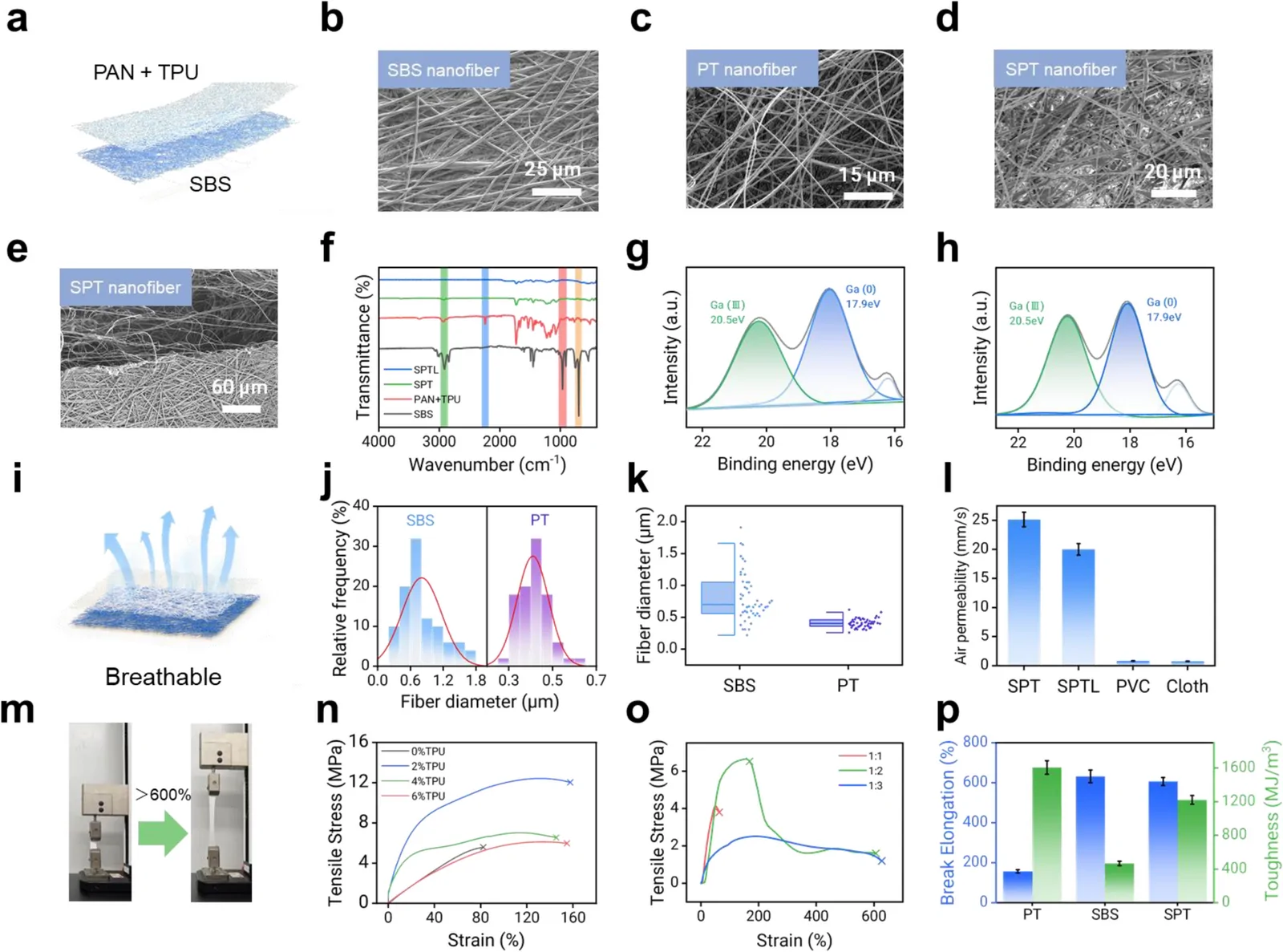
Morphology, chemical composition, breathability, and mechanical properties of the SPTL e-skin. **a** Schematic of SPT structure. SEM of SBS **b**, PT **c**, and SPT **d**. **e** Cross-sectional SEM of SPT. **f** FTIR spectra of SPTL, SPT, PT, and SBS. **g** High-resolution Ga 3*d* spectra of SPTL after scraping off the oxide shell on the LM surface. **h** High-resolution Ga 3*d* spectra of SPTL without scraping off the oxide shell on the LM surface. **i** Schematic of fabric breathability. **j, k** Histogram and half-box plot of the fiber diameter distributions of SBS and PT. **l** Breathability comparison of SPT, SPTL, PVC tape, and cloth tape tested at their respective operational thicknesses. **m** Tensile schematic diagram of SPT. **n** Stress–strain curves of PT with different TPU contents. **o** Stress–strain curves of PT and SBS with different spinning time ratios. **p** Bar chart for mechanical comparison of PT, SBS, and SPT. (In j and k, *n* = 50 randomly selected fibers. In l and p, error bars represent the SD, *n* = 3.)

**Screen Printing of LM Ink:** The final SPTL e-skin was fabricated by patterning the conductive circuit onto the SPT substrate. A 200-mesh screen (opening ≈ 75 μm) was employed, and the prepared LM ink was brushed onto the substrate using a polyurethane scraper. The resulting SPTL device was dried in an oven at 60 °C for 20 min to cure the pattern (Figs. 1, 2).

### Circuit Design of Sensing Modules

**Fig. 3:**
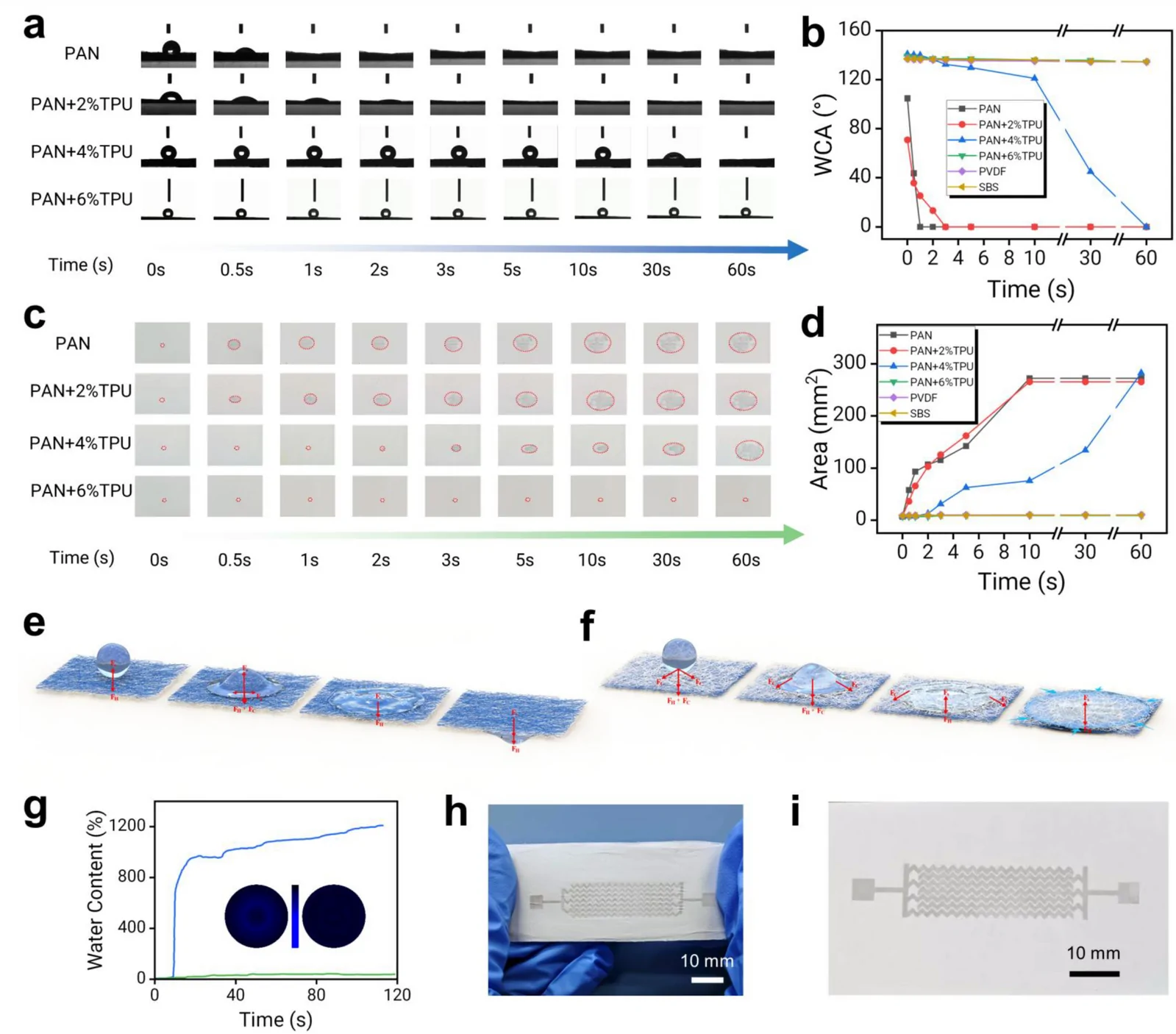
Surface wettability, moisture management, and structural design of the SPTL e-skin. **a** Contact angle evolution of water on the electrospun PAN and TPU with different TPU concentrations. **b** The variation of water contact angles with time on different substrates. **c** Time-lapse images of droplet spreading. **d** Diffusion area as a function of time for water droplet on different material surfaces. **e, f** Proposed water transport mechanism across the hydrophobic (blue, SBS) and hydrophilic (white, PT) layers (*F*_*S*_: hydrophobic force, *F*_*H*_: hydrostatic pressure, *F*_*C*_: capillary force). Although the schematic depicts downward flow, *F*_*C*_ is significantly larger than *F*_*H*_, ensuring active sweat wicking from the skin even against gravity. **g** Dynamic water content curves on the hydrophobic (SBS) and hydrophilic (PT) surfaces measured by MMT; the insets show the corresponding wetting patterns (fingerprints). **h** Tensile-deformation schematic of SPTL-1. **i** Photograph of the SPTL-1 capacitive e-skin with wave-structured electrodes

**Fig. 4:**
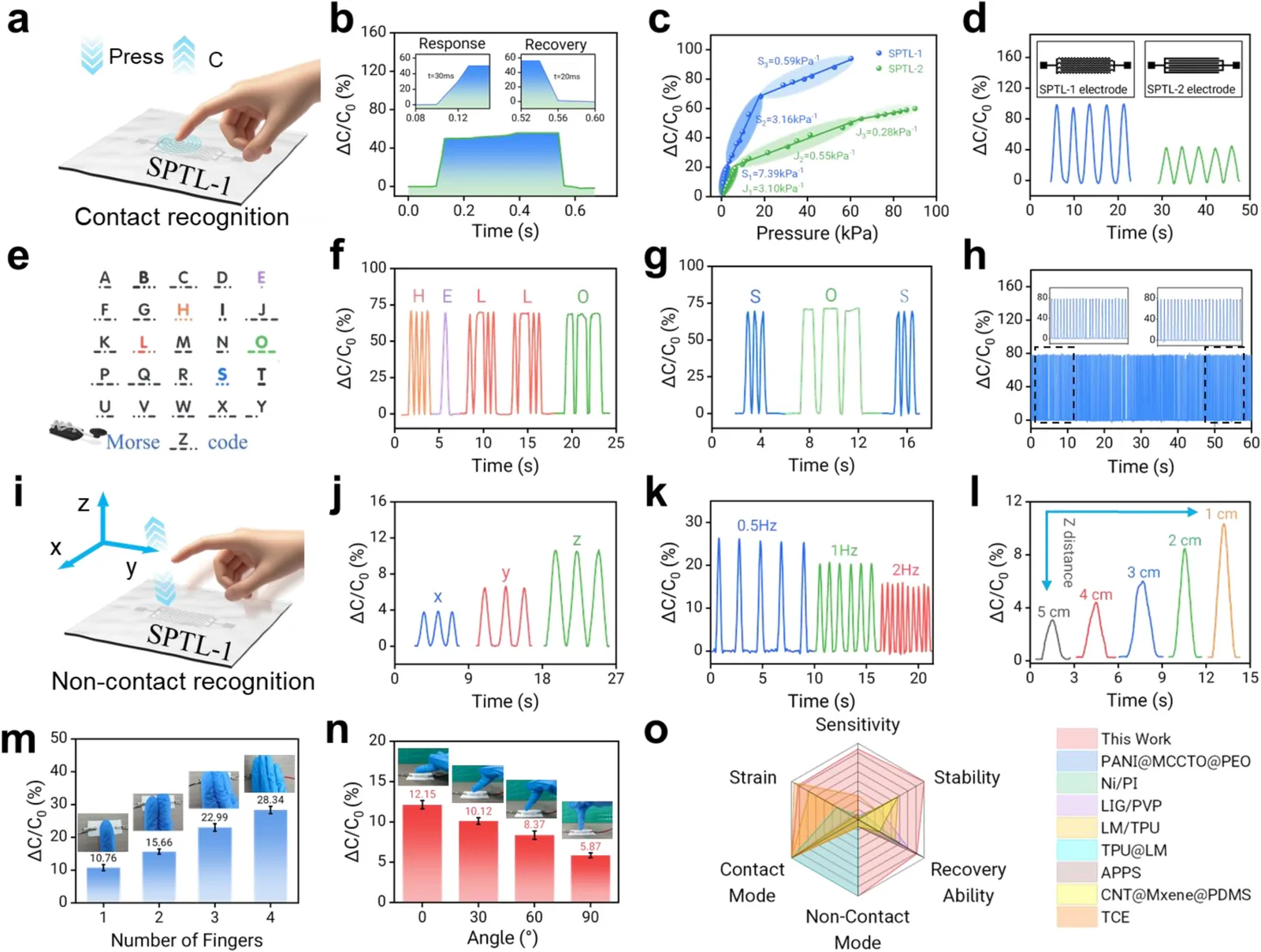
Electromechanical and sensing applications of the SPTL e-skin. **a** Schematic illustrating the relative capacitance change of SPTL-1 under pressure. **b** Pressing response and recovery time of SPTL-1. **c** Pressure sensitivity of SPTL-1 and SPTL-2. **d** Capacitance variation of SPTL-1 and SPTL-2 under the same applied force. **e** Schematic of Morse code letter encoding. **f** Transmitting "HELLO" using Morse code. **g** Transmitting "SOS" via Morse code. **h** Relative capacitance of SPTL-1 during cyclic pressing. **i** Schematic of relative capacitance generated by SPTL-1 in non-contact mode. **j** Relative capacitance response of SPTL-1 to non-contact movement along x, y, z axes. **k** Relative capacitance response of SPTL-1 to non-contact approach at different frequencies (working distance: 1 cm). **l** Relative capacitance changes of SPTL-1 during non-contact approaching at different working distances. **m** Relative capacitance changes of SPTL-1 when different fingers approach in non-contact mode (working distance: 1 cm). **n** Relative capacitance changes of SPTL-1 when fingers approach at different angles in non-contact mode (working distance: 1 cm). **o** Radar chart comparing key parameters between SPTL-1 and other reported devices. (Data are presented as mean ± SD, *n* = 3.)

The specific circuit layouts for the developed e-skins are illustrated in Figs. 3i, 4d, and S18 (later). Specifically, the capacitive e-skins (functioning in contact/non-contact modes) are defined as SPTL-1 (with a wave-shaped electrode structure, Fig. 3i) and SPTL-2 (with a planar straight electrode design, compared in Fig. 4d). The resistive strain e-skin is denoted as SPTL-3 (Fig. S18), while the physiological electrodes are designated as SPTL-4 (EMG/ECG) and SPTL-5 (EEG/EOG), both of which are detailed in Fig. S18.

### Material Characterization

The morphology and microstructure of the fibrous membranes were characterized using a scanning electron microscope (SEM, Regulus 8230) equipped with energy-dispersive X-ray spectroscopy (EDS). Molecular structure was analyzed by Fourier transform infrared spectroscopy (FTIR, NICOLET IS50). Elemental composition and chemical states were investigated using X-ray photoelectron spectroscopy (XPS, PHI QUANTERA-II SXM). The crystallinity of the electrospun membranes was evaluated by X-ray diffraction (XRD, SmartLab SE). To assess surface properties, contact angles and rolling angles were measured using a goniometer (Dataphysics OCA 20), evaluating the wettability, adhesion, and printability of the electrospun films and LM ink. Air permeability was measured with a YG461E-III tester (Ningfang Instrument, China). To evaluate real-world wearability, all measurements were conducted at the actual operational thicknesses of the fabricated membranes (e.g., ~ 30 μm for SPTL) and the commercial control tapes, rather than at an artificially normalized thickness.

The moisture permeability (water vapor transmission rate, WVT) was tested using an RL450 permeability tester (Luozhong, China) at 38 °C, 90% relative humidity, and an airflow velocity of 0.3–0.5 m s^−1^. The WVT was calculated as:

1 $$WVT = \frac{{\left( {\Delta m - \Delta m^{\prime } } \right)}}{At}$$

where WVT is the water vapor transmission rate with the unit of grams per square meter per day (g m^−2 ^ d^−1^); Δ*m*—The difference between two weighings of the same test assembly, with the unit of gram (g); Δ*m*′—The difference between two weighings of the same test assembly for the blank sample, with the unit of gram (g); when no blank test is conducted, Δm′ = 0; A—Effective test area (the device in this part is 0.00283 m^2^), with the unit of square meter (m^2^); *t*—Test time, with the unit of day (d).

Directional liquid transport (one-way conduction) was analyzed using a Moisture Management Tester (MMT, SDLATLAS M290). Mechanical tensile stress–strain curves were obtained using an electronic tensile tester (Mark-10 Instrument Co. Ltd.). Electrochemical impedance spectroscopy (EIS) was performed on an electrochemical workstation (CHI 760E) over a frequency range of 100 kHz to 0.1 Hz. Interfacial contact impedance was measured using electrodes spaced 50 mm apart. Throughout this work, unless otherwise specified, “impedance” refers to the skin-electrode interfacial contact impedance.

To macroscopically visualize the "liquid diode" effect, a standardized penetration test under simulated wearing pressure was designed. Standard qualitative filter papers soaked with blue-dyed aqueous solution were employed as a quantitative moisture source. For the forward anti-gravity pumping test, the membrane was placed on the wet filter papers with the hydrophobic SBS layer facing down, and a glass plate was manually applied on top to simulate standard wearing pressure. Remarkably, the bottom-up fluidic transport initiated instantly, completely saturating the top PT surface within merely 15 s. Furthermore, this active transmembrane pumping persisted continuously throughout the 3-min testing period. Upon uncovering the setup at 180 s, macroscopic water droplets were significantly accumulated on the removed glass slide, definitively confirming the actual transmembrane fluidic pumping. For the reverse anti-leakage test, the membrane (SBS facing down) was placed on top of a pristine dry qualitative filter paper, which served as a strict moisture indicator. Five layers of filter paper soaked with blue-dyed water were then applied onto the upper PT layer. To ensure a strictly controlled comparison, this reverse setup was maintained for the exact same duration (3 min). Subsequently, the setup was disassembled. The underlying indicator filter paper remained perfectly dry and pure white, explicitly confirming a flawless barrier against liquid backflow.

### Physiological Electrical Signal Acquisition System

EMG signals were acquired using SPTL electrodes interfaced with a biological signal acquisition board (OpenBCI, USA). For intensive dynamic testing (e.g., dumbbell lifting), standard breathable medical tape was applied as an auxiliary fixation following standard electromyography protocols to prevent displacement under massive shear forces. Electroencephalogram (EEG) and electrooculogram (EOG) signals were recorded using a three-channel EEG system (Key Laboratory of Brain Health Intelligent Assessment and Intervention, Beijing Institute of Technology). Electrocardiogram (ECG) signals were recorded via an AD2832ECG sensor (SICHIRAY Co., Ltd.). Signal quality was quantified using the signal-to-noise ratio (SNR). The SNR was calculated as follows:

2 $$SNR = 10\log_{10} \frac{{P_{s} }}{{P_{n} }} = 10\log_{10} \frac{{\frac{{\sum\nolimits_{1}^{{N_{s} }} {V_{s}^{2} } }}{{N_{S} }}}}{{\frac{{\sum\nolimits_{1}^{{N_{n} }} {V_{n}^{2} } }}{{N_{n} }}}}$$

where Vs and Vn denote the voltage values of the signal and noise, respectively, and $N_{s}$ and $N_{n}$ represent the number of samples of the signal and noise, respectively.

### Cytotoxicity Assessment

The biocompatibility of the SPT and SPTL films was evaluated using an L929 cell extraction method. L929 cells were seeded in a 96-well plate at a density of $7.0\times 1{0}^{3}$ cells per well and cultured with 100 μL of the film extraction medium at 37 °C in a 5% CO_2_ atmosphere. After incubation periods of 24, 48, and 72 h, 100 μL of CCK-8 solution was added to each well and incubated for an additional 2 h. Optical density at 450 nm (OD450) was measured using a microplate reader (SPARK 10M, TECAN). Live cells were visualized via Calcein-AM staining using laser confocal microscopy (Olympus FV1200). Statistical Analysis: All quantitative data are presented as the mean ± standard deviation (SD). Unless otherwise specified, the number of independent replicates for all experiments (including material characterizations, electromechanical tests, and cell viability assays) is n = 3.

## Results and Discussion

### Structural Design and Characterization of the SPTL E-skin

Figure 1 illustrates the bio-inspired design strategy, fabrication workflow, and application overview of the SPTL e-skin. The *Nepenthes* peristome enables directional water transport from the inner to the outer edge via capillary forces, driving flow from the lower side (α) to the higher side (β) (Fig. 1a). Functionally adapting this macroscopic anti-backflow concept into a transmembrane fluidic design, a bilayer membrane was fabricated by electrospinning, consisting of a hydrophilic PAN-TPU upper layer and a hydrophobic SBS lower layer. PVP was employed as a surfactant to disperse LM into a conductive ink, which was subsequently patterned onto the SBS/PAN-TPU membrane via screen printing (Fig. 1b). The resulting SPTL exhibits high air permeability, waterproofness, unidirectional moisture transport, skin compatibility, and a passive cooling effect (Fig. 1c), enabling high-fidelity electrophysiological signal acquisition and versatile resistive-capacitive sensing. Combining machine learning algorithms, SPTL-based sensing allows recognition of handwritten characters (A-D) and EMG teleoperation of a quadruped robot for information transfer as well as high-risk tasks (Fig. 1d, e).

The SPT structural design with PT as the upper layer and SBS as the lower layer is shown in Fig. 2a. SEM images (Fig. 2b–d) exhibit the micromorphological characteristics of the SBS, PT, and SPT electrospun films. EDS elemental mapping confirms the presence of C in the SBS layer, while the PT layer displays the uniform distribution of C, N, and O, consistent with their chemical compositions. These results align with the inherent elemental species of their respective base materials (Fig. S1). A distinct bilayer architecture of SPT was observed in the SEM cross-section (Fig. 2e). The lower SBS layer exhibits a densely interconnected fiber network, whereas the upper PT layer is relatively loosely arranged, showing distinct microstructural characteristics. Elemental distribution mapping of SPT is presented in Fig. S2. In the cross-sectional mapping of SPT, N, and O are distributed only on the loosely packed side (i.e., the PT layer). This distribution not only enables the identification of SBS versus PT fibers but also confirms that fibers in the dense region originate from SBS, whereas those in the loose region originate from PT. This physical integration without chemical alteration was further corroborated by X-ray diffraction (XRD) analysis (Fig. S3). Figure 2f shows the FTIR spectra of SPTL, SPT, PT, and SBS. PT shows a characteristic stretching vibration peak of the cyano group (-C≡N) near 2240 cm⁻^1^, while SBS exhibits a vibration peak associated with the C = C bond around 966 cm⁻^1^. For SPT, which contains both SBS and PT layers, the intensities of several characteristic peaks are significantly attenuated, whereas the -CH₂- stretching peaks at ~ 2921 and ~ 2846 cm⁻^1^ remain because both PT and SBS contain methylene groups. In contrast, for the SPTL e-skin, the presence of LM hinders effective infrared transmission, leading to the complete disappearance of -CH₂- peaks in the FTIR spectrum.

Figure S4 presents the survey XPS spectra of SPTL with and without scratching of the LM surface oxide layer. The high-resolution Ga 3*d* spectrum was deconvoluted into two peaks assigned to metallic Ga(0) at 17.9 eV and Ga(III) oxide at 20.5 eV. As shown in Fig. 2g, h, during ink preparation, bulk LM is broken into numerous micron-sized particles by ultrasonication. Since small LM particles spontaneously oxidize to form a Ga_2_O_3_ shell (Fig. S5), the steric hindrance effect provided by the mixed system of LM and PVP enables stable dispersion of these small LM particles (Fig. S6). Consistently, Fig. S6 shows that the LM particles in the ink are mainly distributed within the size range of 0–25 μm. Once LM is broken from a bulk phase into fine particles, its specific surface area increases significantly, exposing more surface-active sites to air or the solution medium. As a result, a larger fraction of Ga(0) is oxidized to Ga(III) in Ga_2_O_3_. However, when the oxide layer shell on the LM particles is mechanically scratched, it is not necessary to achieve complete disruption. A sufficient number of ruptured LM particles can re-coalesce, thereby forming a continuous percolating conductive pathway (Fig. S7).

Figure 2i schematically illustrates the air permeability mechanism of SPT. As shown in Fig. 2j, both SBS and PT electrospun fibers exhibit uniform, bead-free morphologies. The SBS fiber diameter ranges from 0.3 to 1.8 µm, while the PT fiber diameter ranges from 0.2 to 0.6 µm, exhibiting higher homogeneity. The half-box plot in Fig. 2k further confirms the centralized fiber-diameter distribution of both samples. The resulting porous fibrous network (Fig. 2i; fiber diameters calculated from Fig. S8) endows SPT and SPTL with air permeabilities of 25.16 and 20.02 mm s^−1^, respectively. These svalues are much higher than the air permeability of polyvinyl chloride (PVC) tape (0.82 mm s^−1^) and cloth tape (0.77 mm s^−1^) tested at their standard functional thicknesses, indicating that the ultra-thin SPTL e-skin (~ 30 μm) possess superior air circulation. In addition, Fig. S9 shows that the moisture permeability of SPTL and SPT is 890.46 and 865.02 g m^−2^ day^−1^, respectively. These values satisfy the physiological requirement for evaporating insensible perspiration (300–600 g m^−2^ day^−1^), thereby supporting skin thermoregulation [37].

Figure 2m schematically illustrates the tensile deformation of SPT, indicating that the bilayer film can sustain large tensile strains. Figure 2n summarizes the mechanical performance of electrospun PT fibers prepared by blending 15 wt% PAN with different TPU contents. The PT fibers exhibit the best overall mechanical properties when the TPU addition ratio is 2 wt%, indicating that an appropriate amount of TPU can improve the stress and strain levels of PAN electrospun fibers. Figure S10 also shows that, at 2 wt% TPU, the tensile strain and toughness of the PT system reach their maximum values, confirming 2 wt% as the optimal TPU concentration. Figure S11 shows that polyvinylidene fluoride (PVDF) exhibits a tensile stress comparable to SBS but a much lower strain. As shown in Fig. 2o, for PT/SBS composites fabricated by electrospinning at different time ratios (1:1, 1:2, and 1:3), the mismatch in stress–strain tolerance between the two layers leads to distinct tensile responses. Specifically, when the time ratio is 1:2, a marked drop in stress is observed during the stretching process. This occurs because the relatively rigid PT layer fractures first, causing a sudden loss of load-bearing capacity, while the highly elastic SBS layer remains intact and continues to stretch up to 606%. By further increasing the SBS content (spinning time ratio of 1:3), the composite film achieves superior stretchability with a maximum tensile strain of 627%. Consequently, the 1:3 ratio was selected for fabricating the SPTL e-skin to ensure optimal deformability. As summarized in Fig. 2p, laminating PT (low strain) with SBS (low toughness) yields an SPT film that simultaneously achieves high stretchability and improved toughness. It is worth noting that the structural integrity of this Janus dual-layer is intrinsically guaranteed by the sequential electrospinning process. As conceptually illustrated in Fig. 1c, the PAN-TPU fibers are directly deposited onto the freshly spun SBS network. This creates robust physical interlocking and micro-entanglement at the interface, effectively preventing delamination even under extreme mechanical deformation.

The cooling performance in Fig. S12 indicates that the SPT maintains a lower temperature than commercial Ag/AgCl electrodes during both daily wear and exercise. This pronounced passive cooling effect is a direct thermodynamic consequence of the Janus liquid diode mechanism, where the rapid evaporation of actively wicked sweat efficiently dissipates the latent heat of vaporization. The cytotoxicity tests in Fig. S13 show no significant difference in cell numbers between the SPT/SPTL groups and the control, indicating negligible cytotoxicity and good cytocompatibility. To further assess in vivo biosafety, a 2-day forearm skin-adhesion test was performed with SPTL, cloth tape, PVC tape, and Ag/AgCl electrodes (Fig. S14). PVC tape and Ag/AgCl electrodes induced evident erythema after removal, likely due to irritation or mechanical damage associated with poor biocompatibility and excessive adhesion, whereas cloth tape caused only mild redness. In contrast, the SPTL e-skin produced no observable adverse skin reactions, confirming excellent skin compatibility.

### Unidirectional Moisture Transport and Liquid Diode Mechanism

Figures 3a, b and S15a show the time-dependent contact angle images of 3 μL DI water droplets on PAN-TPU films with different TPU concentrations, as well as on PVDF and SBS electrospun films. PAN blended with 0% or 2% TPU exhibits good hydrophilicity, whereas increasing the TPU content to 4% and 6% markedly reduces surface hydrophilicity. In contrast, both pristine SBS and PVDF electrospun films are distinctly hydrophobic. Figures 3c, d and S15b further quantify droplet spreading via the time evolution of the droplet-substrate contact area. Hydrophilic surfaces show a pronounced increase in contact area over time, while hydrophobic surfaces exhibit only minor changes. These trends agree with the contact-angle measurements, confirming that film composition governs the wettability of the electrospun membranes. Figure S16 provides contact-angle images of blue ink on PT and SBS, visually highlighting the wettability contrast between the two surfaces. Figure 3e, f conducts a macroscopic force analysis of directional moisture transport in the Janus moisture-wicking film. The mechanism underlying this "liquid diode" effect is fundamentally driven by the asymmetric capillary forces and Laplace pressure gradients established by the wettability gradient between the Janus layers. When the SBS nanofiber layer faces upward, the droplets contacting this layer will be subjected to an upward hydrophobic force (F_S_), which partially counteracts the downward hydrostatic pressure (F_H_). As the capillary force (F_C_) generated by the PT nanofibers increases, it eventually overcomes the hydrophobic force (F_S_) and breaks the transport barrier, prompting the droplets to penetrate into the underlying hydrophilic layer. It is worth noting that due to the dominance of F_C_ at this micro-scale, the device maintains effective directional transport even against gravity (e.g., vertical wicking). Once inside, the strong and sustained F_C_ continuously draws droplets through the SBS layer into the superhydrophilic network, culminating in liquid exudation from the superhydrophilic PAN nanofibers. On the contrary, when the film is flipped (Fig. 3f), the droplets are immediately dominated by the omnidirectional capillary force (F_C_) from the PAN nanofibers, leading to rapid wetting until the superhydrophilic layer approaches saturation. Subsequently, the hydrophobic SBS nanofibers provide an upward surface tension (F_S_), which acts as a robust energy barrier in the absence of a reverse capillary driving force, effectively blocking further liquid penetration and thereby preventing dripping [38]. Overall, this force balance elucidates the underlying mechanism by which the SPTL e-skin achieves unidirectional moisture transport. To visually corroborate this theoretical model, macroscopic penetration experiments under simulated pressure were conducted (Fig. S17), explicitly confirming the robust anti-gravity sweat pumping and absolute reverse anti-leakage capabilities.

As shown in Fig. 3g, the dynamic liquid transport capability of the SPT membrane was evaluated using a Moisture Management Tester (MMT). Upon dropping simulated sweat on the hydrophobic SBS side, the water content on the SBS surface (inner layer) remains at a low level, whereas the water content on the hydrophilic PT surface (outer layer) increases rapidly and maintains a high level. This significant difference, visually confirmed by the wetting patterns (fingerprints) in the insets, demonstrates that sweat is effectively pumped from the inner side to the outer side, preventing backflow. Quantitatively, the SPT film exhibits a high cumulative one-way conduction capacity of 956.36 and an overall liquid moisture management capacity (OMMC) of 0.62. Achieving these outstanding moisture transport metrics on a structurally optimized membrane (the 1:3 ratio) confirms an ideal practical balance between indispensable mechanical wearability (i.e., 627% stretchability) and active sweat management. These values not only meet practical requirements for wearable monitoring but also significantly outperform reported benchmarks (e.g., 696.63 in Ref. [39]), demonstrating superior unidirectional liquid transport capability. Figure 3h demonstrates the SPTL e-skin under tensile deformation, while Fig. S18 further illustrates its structural integrity under other mechanical distortions (bending, squeezing, and twisting). SPTL maintains structural integrity, high flexibility and mechanical robustness. Figures 3i and S19 jointly demonstrate the printability of SPT. Figure 3i presents a screen-printed capacitive e-skin (SPTL-1) fabricated with LM ink. Notably, beyond the enhanced capacitive sensitivity, the wave-structured electrode design is specifically engineered to accommodate macroscopic tensile deformation [40]. Unlike planar straight electrodes (SPTL-2) that are prone to mechanical fracture under massive tension, the wavy architecture can effectively unfold to dissipate localized stress. This structural design perfectly complements the intrinsic ultrahigh stretchability (627%) of the SPT substrate, ensuring superior electrical and mechanical stability under large strain. Figure S20 further shows the diversified screen printing results, and this process enables high-resolution printing down to 250 μm.

### Contact and Non-Contact Multimodal Sensing Capabilities

LM exhibits an extremely high surface tension (624 mN m⁻1) [41], causing pristine LM ink to coalesce rather than adhere to the SPT substrate. Introducing the surfactant PVP during ink formulation can temporarily suppress droplet re-aggregation, markedly enhancing processability and facilitating pattern miniaturization [42]. As shown in Fig. S21, compared with pristine LM, the LM ink exhibits a smaller contact angle, showing better fluidity, wettability and printability. Meanwhile, the rolling angle of the LM ink is larger than that of pure LM (Fig. S22), suggesting stronger adhesion on the SPT. Figure S23 shows that the electrical conductivity of SPTL is 0.47 MS m^−1^. To evaluate the deformation resistance of the resistive e-skin SPTL-3, the effects of three typical mechanical deformations (bending, twisting, and stretching) on its relative resistance were investigated in Fig. S24. The results show that under bending or twisting from 0° to 180°, the relative resistance remains stable without obvious changes. In contrast, during stretching, the circuit structure is progressively damaged as strain increases, resulting in a pronounced resistance change. For dynamic performance evaluation, SPTL-3 was worn on a finger knuckle. In Fig. S25, different knuckle bending angles produce different amplitudes of relative resistance change, and larger bending angles lead to larger resistance variations. In addition, SPTL-3 exhibits excellent dynamic responsiveness during finger bending, with a response time of 30 ms and a recovery time of 20 ms, enabling rapid capture of finger joint motion (Fig. S26), which is essential for constructing multifunctional HMI.

Figure S27 explains the mechanism of relative capacitance change in the capacitive e-skin SPTL-1 under two working modes: contact and non-contact. In the contact mode, a finger directly contacts and enters the electric field between the two opposite electrode plates, causing the relative capacitance changes by altering the dielectric constant between the electrode plates. In the non-contact mode, the finger does not touch the device but moves through the fringing electric field above the electrode plates. The dielectric-constant change in the region occupied by the finger likewise induces a relative capacitance variation. Notably, because the electric field intensity outside the two plates is much weaker than that in the facing region between them, the capacitance contribution from the non-contact mode effect is negligible during pressing (i.e., in contact-mode operation). Figure S28 further describes the capacitance characteristics of interdigital electrodes, providing an intuitive basis for understanding their capacitance behavior and guiding subsequent e-skin design [43, 44].

Figure 4a schematically illustrates the relative capacitance change of the capacitive e-skin SPTL-1 under pressing. In Fig. 4b, SPTL-1 exhibits an ultrafast dynamic response, with a response time of 30 ms and recovery time of 20 ms. In terms of sensitivity, SPTL-1 outperforms SPTL-2. The sensitivity of SPTL-1 in the low-pressure range reaches 7.39 kPa⁻1, enabling it to accurately capture weak pressure signals (Fig. 4c). To clarify the capacitance disparity between SPTL-1 and SPTL-2 and establish a quantitative relationship, a theoretical analysis was conducted based on the geometric model in Fig. S29. Let $d_{3}$ denote the electrode thickness. Based on the geometric structure, other parameters are defined as follows: x is the unit width of the wave structure, $d_{4}$ is the wave height, $d_{1}$ is the planar spacing between electrodes, and α is the structural inclination angle. According to the geometric relationship where $tan \alpha = \frac{{2d_{4} }}{x}$, a theoretical analysis was conducted based on the geometric model and detailed derivation presented in Fig. S29, yielding the following capacitance ratio:

3 $$\frac{{{\mathrm{C1}}}}{{{\mathrm{C2}}}}{ = }\sec^{2} \alpha - \frac{{2d_{1} \tan \alpha }}{x} = \tan^{2} \alpha - \frac{2d1}{x}\tan {\upalpha } + 1 = 1 + \frac{{4d_{4} }}{{x^{2} }}\left( {d_{4} - d_{1} } \right)$$

Since the wave height $d_{4}$ is designed to be larger than the spacing $d_{1} \left( {d_{4} > d_{1} } \right)$, it follows that $\frac{{C_{1} }}{{C_{2} }} > 1$. This theoretical derivation confirms that the wave-structured electrode design of SPTL-1 inherently provides a larger effective capacitance compared to the planar electrode configuration of SPTL-2. Figure 4d experimentally verifies this, showing that SPTL-1 exhibits a consistently higher relative capacitance than SPTL-2 under the same applied force, which corroborates the above calculation.

Functional application tests of SPTL-1 demonstrated the transmission of Morse code (Fig. 4e–g), confirming its capability to recognize specific pressing actions. To further verify the dynamic response and reliability, the SPTL-1 e-skin was used to input the Morse code for "Hello", which was accurately decoded and displayed on the software interface in real time (Movie S1). Performance differentiation tests indicated that under the same contact area and pressure, pressing SPTL-1 with different materials produces distinct relative capacitance changes, suggesting feasibility for material identification (Fig. S30). Furthermore, when the material and contact area are fixed, the applied pressure is positively correlated with the relative capacitance change, enabling pressure-level discrimination (Fig. S31). Dynamic performance and durability tests further confirmed the reliability of SPTL-1. In frequency adaptability tests (1–5 Hz), the relative capacitance change amplitude remained stable, and only the change speed increased with frequency (Fig. S32). After 60 s of continuous pressing, the relative capacitance change maintained good consistency (Fig. 4h). After 10,000 pressing cycles, the initial relative capacitance decayed by less than 3%, demonstrating excellent fatigue resistance and the operational stability of the activated LM conductive network (Fig. S33). Meanwhile, SPTL-1 demonstrates outstanding capability for detecting weak signals. A 0.15 g lightweight object produces a clear capacitance response, and when attached to the throat region, the SPTL e-skin can capture subtle deformations associated with swallowing (Fig. S34). These results highlight its strong potential for micro-force and micro-deformation monitoring applications, which is critical for precise tactile sensing and motion monitoring.

Figure 4i schematically illustrates how non-contact interactions influence the relative capacitance change of SPTL-1. In practical applications, the signal sources from contact and non-contact modes can be reliably distinguished based on their distinct waveform profiles and amplitude thresholds (Fig. S35). Specifically, pure non-contact movements generate relatively low capacitance peaks. Notably, during a continuous approaching-to-pressing action, the initial non-contact phase exhibits a precursor plateau whose amplitude perfectly corresponds to the pure non-contact peaks. Following this precursor signal, the actual contact mode (pressing) generates a significantly sharper and higher-amplitude capacitance peak driven by macroscopic physical deformation. The SPTL e-skin exhibited direction-dependent responses during motion along the x, y, and z axes (Fig. 4j). At a fixed working distance of 1 cm (Fig. 4k), tests at different frequencies revealed that the relative capacitance change amplitude decreased with increasing frequency, whereas the response rate accelerated. In Fig. 4l, reducing the working distance of SPTL-1 produced larger capacitance changes. Moreover, at a 1 cm working distance, increasing the number of fingers (i.e., contact area) enhanced the capacitance change amplitude (Fig. 4m), and varying the approach angle revealed that greater parallelism between the fingers and the SPTL e-skin surface yielded a more pronounced response (Fig. 4n). Figure S36 further shows that approach speed substantially affects the signal peak width in non-contact mode. Finally, a comparative analysis against e-skins reported in the literature (Table S1) shows that SPTL-1 exhibits markedly superior overall performance across key metrics, including sensitivity, response time, durability, non-contact and contact detection capability, and strain range (Fig. 4o).

### High-Fidelity Electrophysiological Signal Monitoring

**Fig. 5:**
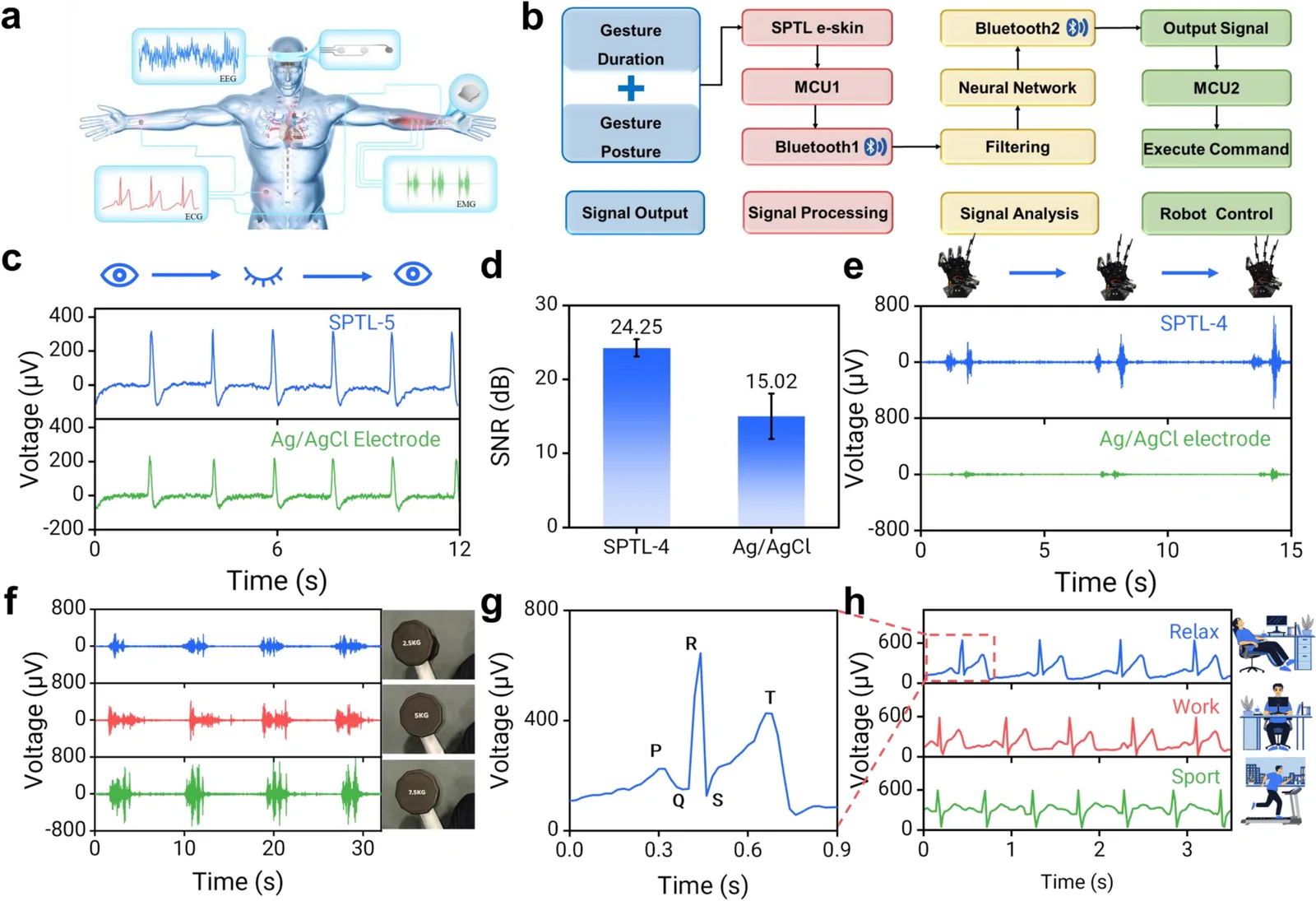
Applications of SPTL e-skins in electrophysiological signal monitoring and HMI. **a** Schematic of electrophysiological signal monitoring (EEG, ECG and EMG). **b** Schematic of the quadruped robot control workflow. MCU: Microcontroller Unit. **c** EOG recordings during eye-blinking at rest. **d** SNR comparison of EMG signals measured using the SPTL-4 electrode and an Ag/AgCl electrode. **e** EMG recordings acquired by the SPTL-4 electrode and an Ag/AgCl electrode to control the manipulator for executing "1", "2", and "3" movements. **f** EMG signals acquired by the SPTL-4 electrode during dumbbell lifting at different loads. **g, h** Collected ECG signals under different physiological states. (Error bars represent the SD, *n* = 3.)

Figure 5a illustrates the schematic of electrophysiological signal monitoring (EEG, EMG and ECG). Figure 5b schematically depicts the EMG teleoperation workflow for a quadruped robot. Briefly, gesture-dependent temporal and intensity features are first captured by the EMG acquisition system and converted into electrical signals. These signals are transmitted via Bluetooth to a machine-learning module for recognition and classification. The resulting control commands are subsequently sent to the quadruped robot, where an onboard ESP32 controller actuates eight servos (Fig. S37) to execute the corresponding motion. To investigate the temporal stability of SPTL-4 (Fig. S38), electrodes with their surface oxide layer scraped off were stored for 1, 3, and 7 days. Their Bode plots, impedance, EMG recordings, and corresponding SNR were evaluated against Ag/AgCl electrodes. The results show that SPTL-4 is significantly superior to Ag/AgCl electrodes in impedance stability, signal stability, and EMG SNR. While the metal layer exposed after scraping off the oxide layer gradually re-oxidizes, this re-oxidation is inherently self-limiting. As a result, it leads to a slightly lower SNR on day 3 compared to day 1, but the surface oxidation tends to stabilize thereafter, resulting in only minor SNR fluctuations. This indicates excellent long-term storage stability for SPTL-4. In contrast, commercial Ag/AgCl electrodes rely on hydrogels that deteriorate over time, causing continuous performance degradation. Additionally, although SPTL-5 may also undergo LM oxidation, its impedance and SNR remain substantially better than those of Ag/AgCl electrodes.

To explore the influence of sweat on electrode signals (Fig. S39), the physiological environment of heavy sweating was simulated by applying 3 mL of artificial sweat to the skin. Under these conditions, SPTL-4 exhibited significantly better impedance stability, signal stability, and EMG SNR compared to the Ag/AgCl electrodes. By actively pumping bulk sweat away from the skin side via the "liquid diode" effect, the SPTL ensures a profoundly dry interface. Physically eliminating the accumulation of interfacial liquid eradicates the root cause of parasitic ion-conductive pathways, impedance fluctuation, and signal cross-talk. To further validate this operational robustness under realistic dynamic wear, a volunteer wore the SPTL-4 e-skin during prolonged dynamic exercises, including a 30-min intensive spinning bike exercise and a 15-min continuous dumbbell lifting exercise. As shown in Fig. S40, the electromyogram (EMG) signals were continuously monitored over the entire 1800 and 900 s periods, respectively. Magnified insets extracted from the early and late stages of both exercises demonstrate that the e-skin consistently captured high-fidelity EMG signals with consistent burst amplitudes and a stable baseline despite profuse natural sweating and continuous mechanical deformation, thereby confirming its highly reliable long-term performance in practical scenarios. Blinking monitoring in a relaxed state showed that the EOG signal amplitude recorded with the SPTL-5 electrode was significantly higher than that of the commercial Ag/AgCl electrode (Fig. 5c). Moreover, the SPTL e-skin demonstrated the capability to capture weak neural activities, enabling stable, long-term acquisition of high-fidelity EEG alpha rhythms at 10 Hz (Fig. S41). In gesture-based manipulator control tasks (“1”, “2”, and “3”), EMG signals acquired using the SPTL-4 electrode exhibited higher intensity than those from the Ag/AgCl electrode, with a markedly improved signal-to-noise ratio (24.25 vs. 15.02 dB), confirming the superior acquisition quality of SPTL-4 (Fig. 5d, e). To further verify the signal response characteristics, EMG signals were recorded while the subject lifted dumbbells of 2.5, 5, and 7.5 kg. As shown in Fig. 5f, the EMG amplitude increased with dumbbell weight, indicating that SPTL-4 can sensitively resolve changes in muscle-activity intensity. Furthermore, accurate acquisition of continuous ECG signals is critical for early screening and diagnosis of cardiovascular diseases [45]. By analyzing heart rate, waveform morphology, and related ECG features, arrhythmia can be effectively identified, helping to prevent irreversible organ damage and safeguard patient safety [46]. The SPTL-4 electrode attached to the skin can clearly capture the characteristic PQRST complexes (Fig. 5g, h) and reliably differentiate ECG patterns under different physiological states. These waveform features enable the assessment of heart rate and overall cardiac condition, providing important technical support for telemedicine-oriented cardiac health monitoring [47].

### Machine Learning-Enabled Human–Machine Interaction and Teleoperation

**Fig. 6:**
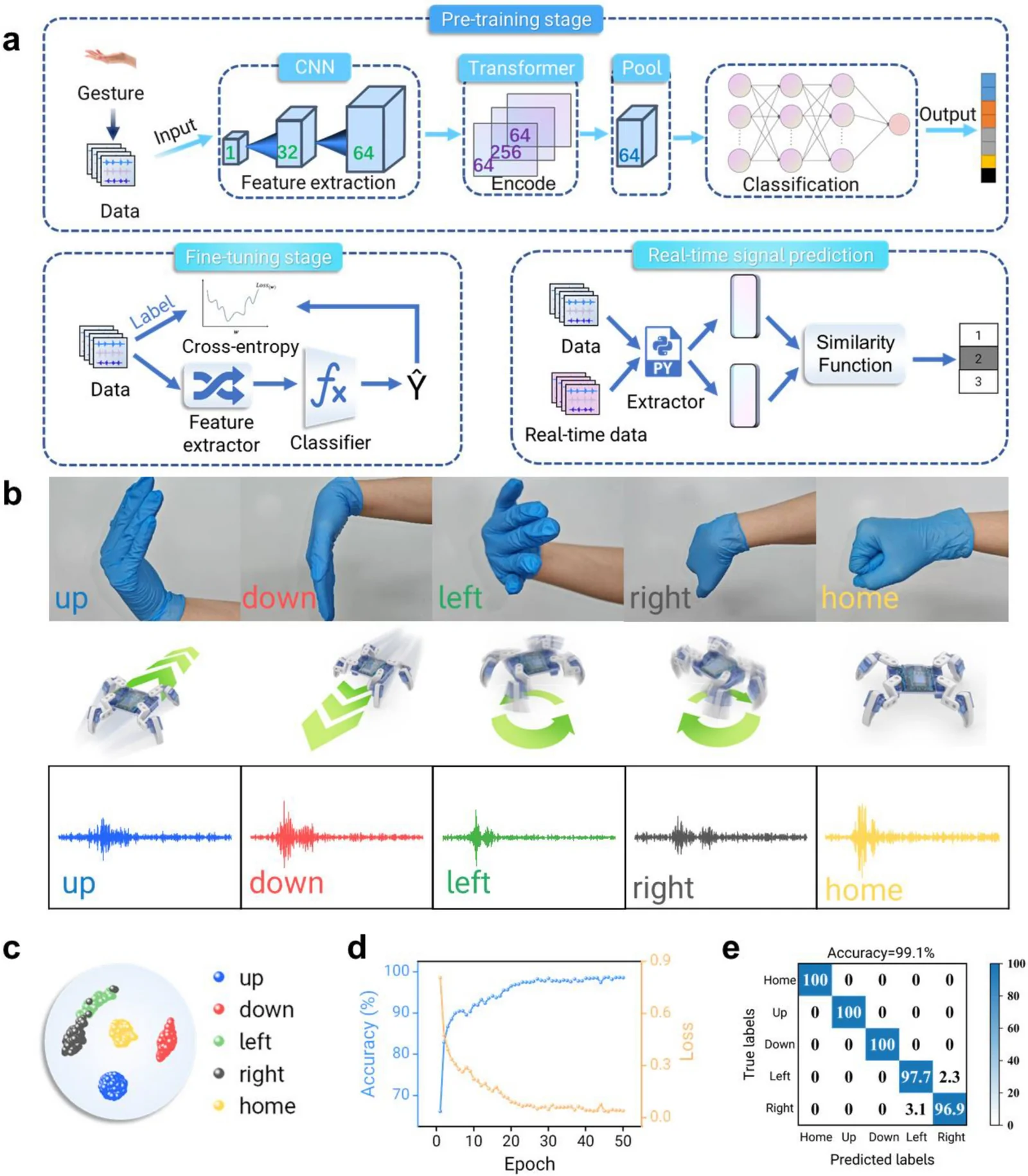
Machine learning-enabled EMG teleoperation of the quadruped robot. **a** Flowchart of machine learning for the EMG-based teleoperation system. **b** Quadruped robot movement modes and the corresponding EMG control signals. **c** t-SNE visualization of the high-dimensional latent features for different commands. **d** Accuracy and loss curves during the fine-tuning stage. **e** Confusion matrix for teleoperation command classification

Figure 6a illustrates the data preprocessing and machine learning pipeline. Raw SPTL e-skin signals generated by user gestures (e.g., "up", "down", "left", "right", "home") are first collected and stored. Subsequently, a custom data processing module executes a sequence of cleaning operations: removing NaN and infinite values, trimming the initial and final 5 s of data to eliminate artifacts, and applying a Butterworth band-pass filter (0.1–50 Hz) for noise reduction. Finally, the data is normalized to a standardized range using a scaling algorithm. The processed dataset, including e-skin data and corresponding gesture labels ("home": 0, "up": 1, "down": 2, "left": 3, "right": 4), is saved for model training. During the training and prediction stages, a sliding window (default size: 32) segments the time-series data, which is then fed into a lightweight Transformer encoder model to extract high-dimensional time-series features. For real-time inference, the prediction module maintains data buffers to perform real-time preprocessing and feature extraction. To ensure robustness, a majority voting mechanism is employed to stabilize the prediction results. A fine-tuning strategy based on transfer learning was adopted to adapt the pre-trained model to specific tasks and diverse user habits. By collecting a small dataset of labeled data, a linear layer classifier was integrated at the end of the encoder for supervised fine-tuning. To rigorously evaluate the model and mitigate overfitting, the dataset was partitioned into training (80%), validation (10%), and testing (10%) sets, with an early stopping strategy implemented by continuously monitoring the validation loss. Furthermore, to address inherent inter-individual physiological variations, the framework was deployed as a highly accurate subject-specific personalized model. This process effectively normalizes variations in movement duration and intensity within the targeted user.

Figure 6b displays the five control gestures alongside the corresponding robot motion schematics and EMG signals. The real-time acquisition and synchronous display of these high-fidelity signal waveforms and hand motions are dynamically demonstrated in Movie S2. To visualize the feature separation capabilities, t-distributed StochasticNeighbor Embedding (t-SNE) was used to project the high-dimensional latent features into a 2D space (Fig. 6c), revealing distinct clusters for different gestures. The efficacy of the fine-tuning process is evidenced by the accuracy and loss curves in Fig. 6d, where the loss drops to 0.04 and accuracy exceeds 99% after only 50 epochs. The confusion matrix (Fig. 6e) further confirms this high performance, showing 100% accuracy for "up/down" and "home" gestures, with less than 3% confusion between "left" and "right". Based on this high recognition accuracy, the decoded EMG signals were wirelessly transmitted to the quadruped robot, enabling precise and low-latency teleoperation in unstructured environments, as demonstrated by the synchronized movements of the operator and the robot (Movie S3). Additionally, the versatility of the proposed e-skin was evaluated on a handwritten letter recognition task (A, B, C, D). As shown in Fig. S42, the SPTL-4 e-skin achieved a recognition accuracy of over 95%, supported by clear t-SNE clustering and rapid convergence of the loss function.

## Conclusions

In summary, we have successfully created a Nepenthes-inspired intelligent e-skin to tackle the sweat accumulation and signal instability during prolonged use. By employing electrospinning to construct a Janus bilayer architecture with an asymmetric wettability gradient, the device achieves autonomous and unidirectional sweat transport via a "liquid diode" mechanism, effectively preventing liquid backflow and maintaining a dry e-skin-skin interface. The SPTL exhibits a high tensile strain of 627%, superior breathability (20.02 mm s^−1^), and a pressure sensitivity of 7.39 kPa^−1^. Crucially, the device demonstrates versatile multimodal sensing capabilities, specifically with the SPTL enabling precise bending-resistive monitoring, non-contact capacitive detection, and robust pressure-capacitive sensing for real-time Morse code communication. Consequently, it allows for the high-fidelity recording of electrophysiological signals (EEG, EMG, and ECG) that significantly surpass commercial Ag/AgCl electrodes. Furthermore, by integrating the e-skin with machine learning algorithms, high-precision EMG-based teleoperation of a quadruped robot and handwriting recognition were successfully achieved with accuracies exceeding 95%. This work provides a promising strategy for designing breathable, robust, and intelligent bio-integrated interfaces for next-generation healthcare and HMI systems.

## Supplementary Information

Below is the link to the electronic supplementary material.

Supplementary file1 (DOCX 10388 KB)

Supplementary file2 (MP4 1545 KB)

Supplementary file3 (MP4 3821 KB)

Supplementary file4 (MP4 3486 KB)
