# Supplementary material for: Intelligent Breathing Electronic Skin Inspired by Nepenthes for Active Sweat Management, Multimodal Sensing and High-Fidelity Electromyographic Teleoperation Using Machine Learning
Source: Nanomicro Lett. 2026 Jun 12;18:401. doi: 10.1007/s40820-026-02252-2 (PMC13263376; doi:10.1007/s40820-026-02252-2)
Supplement: Supplementary file 1 — Supplementary file1 (DOCX 10388 KB) [file 40820_2026_2252_MOESM1_ESM.docx]

Supporting Information for

**Intelligent Breathing Electronic Skin Inspired by *Nepenthes* for Active Sweat Management, Multimodal Sensing and High-Fidelity Electromyographic Teleoperation Using Machine Learning**

Yichen Li^1,2^, Kai Zheng^1,2^, Guangtian Zhang^1,2^, Wentao Chen^1,2^, Chengtan Liu^1,2^, Seonho Shin^1,2^, Bihai Yang^1,2^, Ran Cai^1,2,^*, Bin Hu^1,2,^*

^1^School of Medical Technology, Beijing Institute of Technology, Beijing 100081, P. R. China

^2^Key Laboratory of Brain Health Intelligent Evaluation and Intervention, Ministry of Education, Beijing Institute of Technology, Beijing 100081, P. R. China

*Corresponding author. E-mail: [bh@bit.edu.cn](mailto:bh@bit.edu.cn) (Bin Hu); [cairan@bit.edu.cn](mailto:cairan@bit.edu.cn) (Ran Cai)

**Supplementary Figures and Tables**


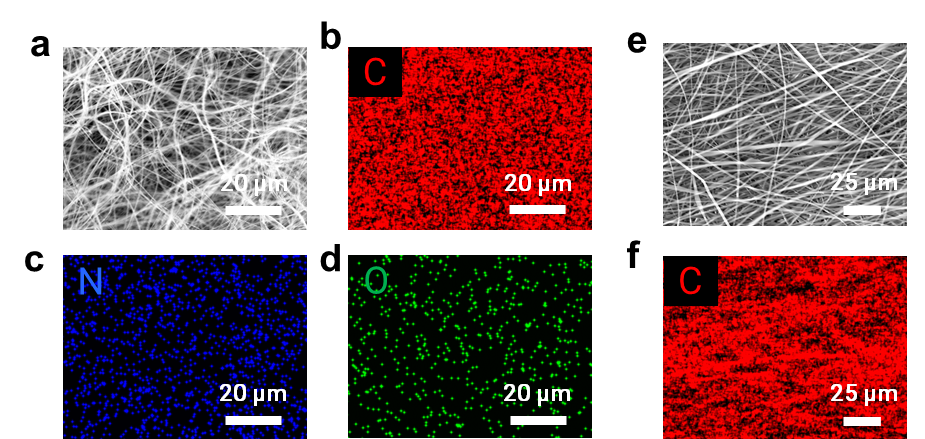


**Fig. S1 Elemental mapping of PT and SBS nanofibers.** (a) SEM image of PT nanofibers. (b–d) Elemental mapping of C (b), N (c), and O (d) for PT. (e) SEM image of SBS nanofibers. (f) Elemental mapping of C for SBS.


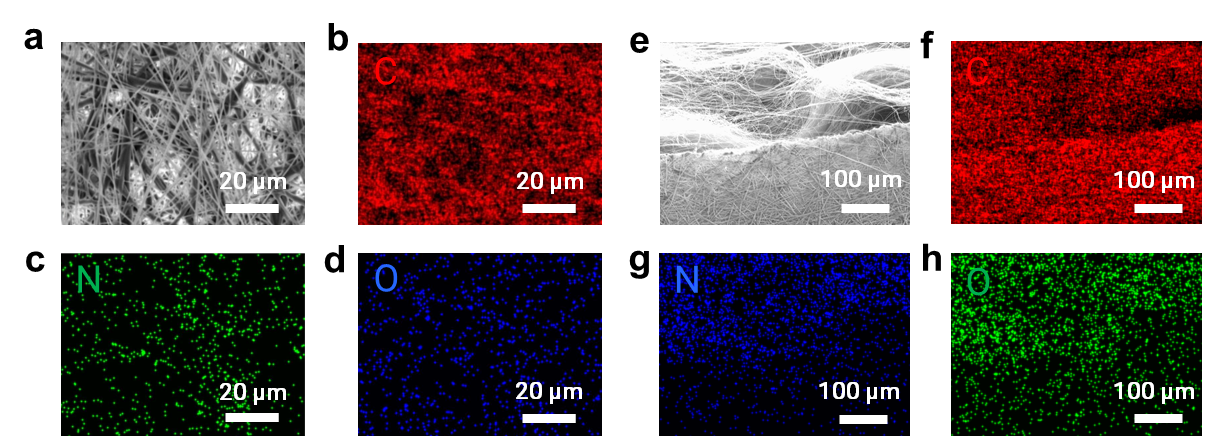


**Fig. S2 SEM and EDS characterization of the SPT e-skin surface and cross-section.** (a) Surface SEM image of SPT. (b–d) Surface elemental mapping of C (b), N (c), and O (d). (e) Cross-sectional SEM image of SPT. (f–h) Cross-sectional elemental mapping of C (f), N (g), and O (h).


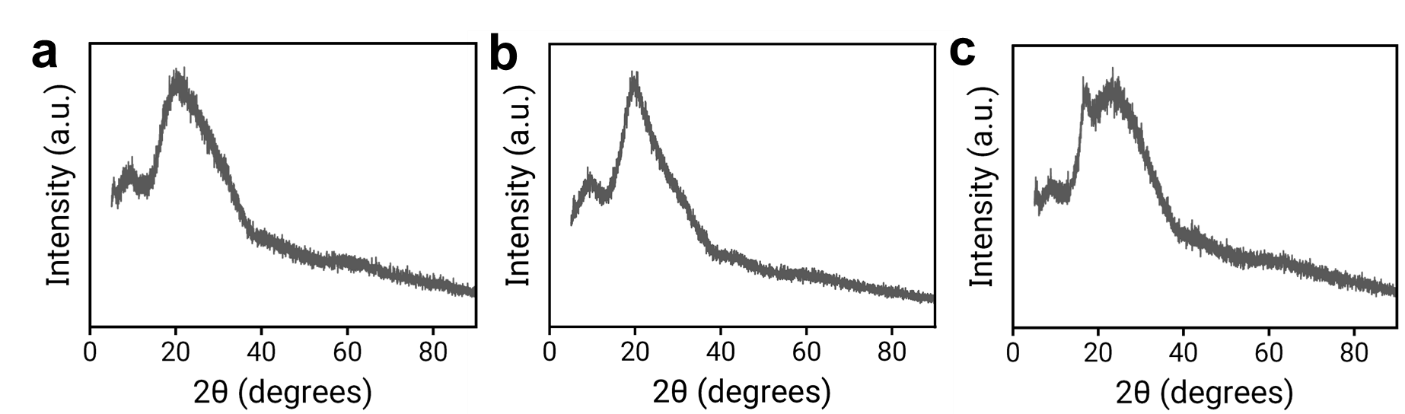


**Fig. S3 Crystallographic structure analysis of the e-skin components**. X-ray diffraction (XRD) patterns of SPT (a), SBS (b), and PT (c).


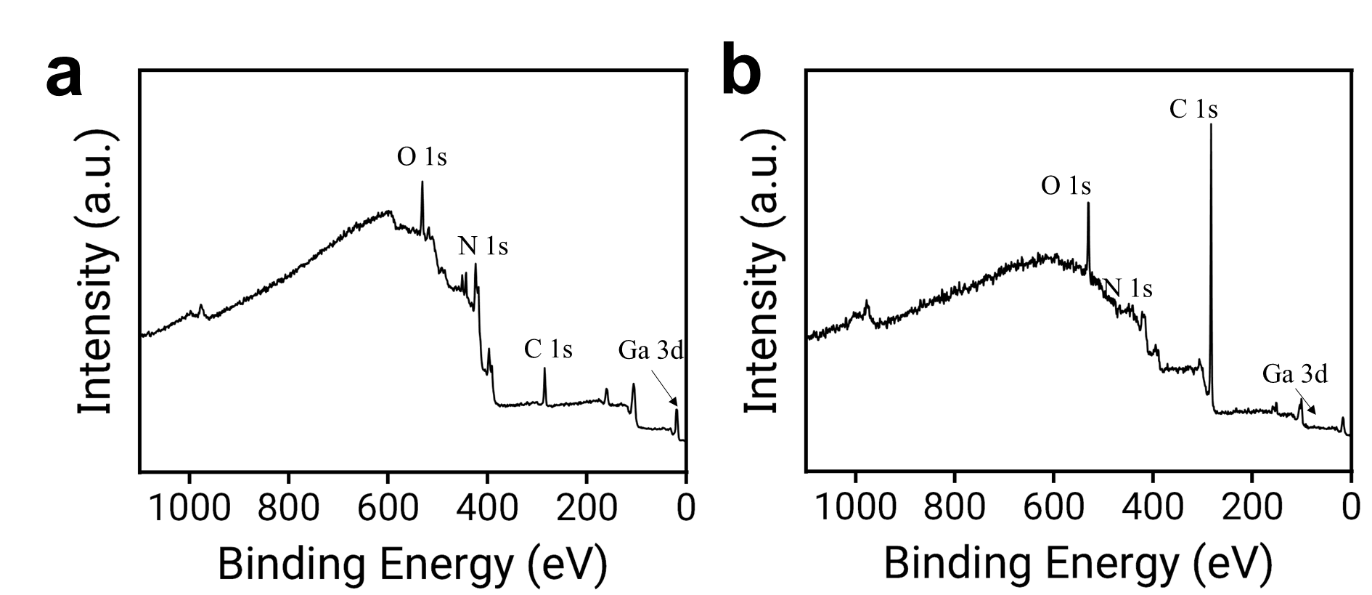


**Fig. S4 XPS survey spectra of the SPTL e-skin.** (a) XPS survey spectrum of SPTL after scraping off the oxide layer on the LM surface. (b) XPS survey spectrum of SPTL without scraping off the oxide layer on the LM surface.


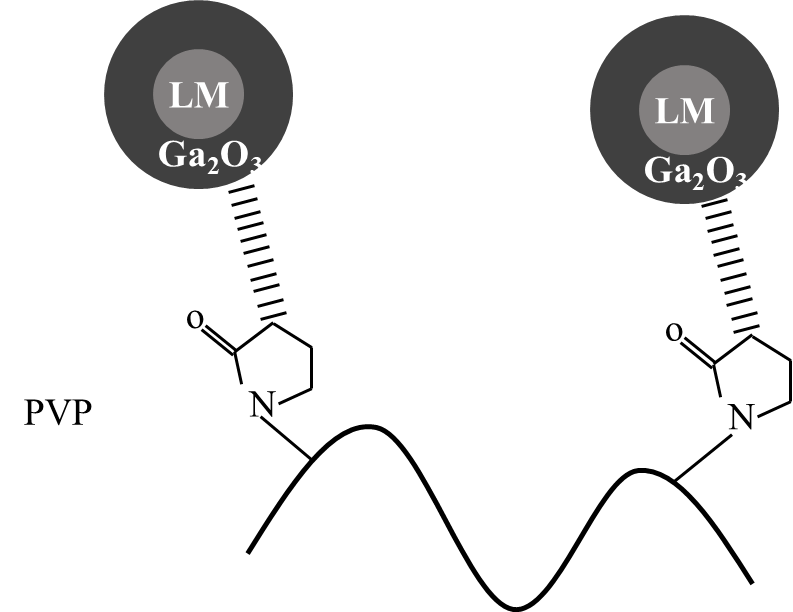


**Fig. S5 Dispersion mechanism of LM particles.** Schematic diagram illustrating the principle of LM dispersion into small particles under the steric hindrance effect.


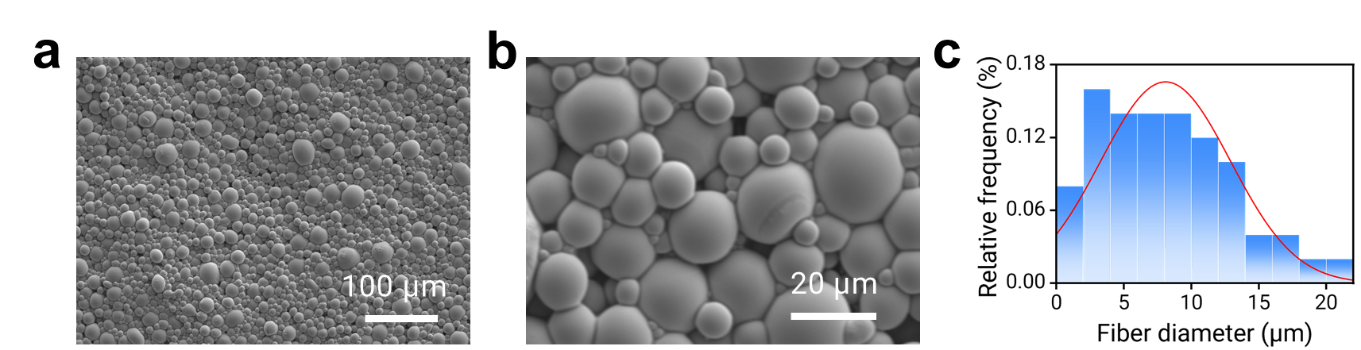


**Fig. S6 Morphology and size analysis of LM/PVP particles.** (a), (b) SEM characterization images and (c) the corresponding particle size distribution histogram of the LM/PVP mixture.
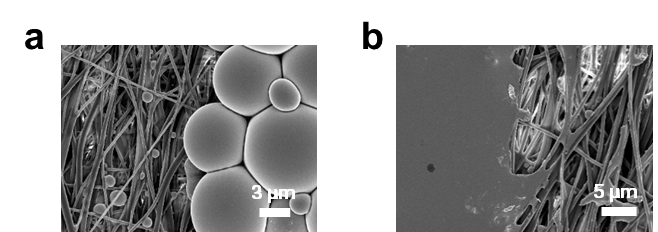


**Fig. S7** **Surface morphology of the LM ink printed on SPTL.** SEM images showing the surface (a) without scraping off the oxide shell on the LM particles, and (b) after scraping off the oxide shell to form conductive paths.


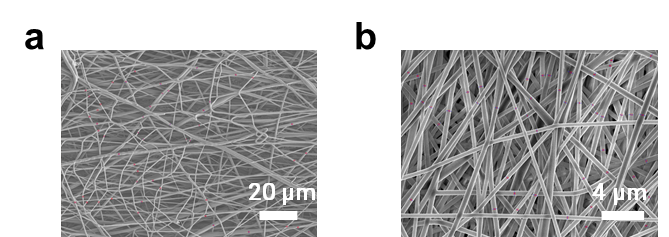


**Fig. S8 Fiber-diameter characterization of the nanofibrous membranes.** SEM images of SBS (a) and PT (b) used for fiber-diameter calculation.


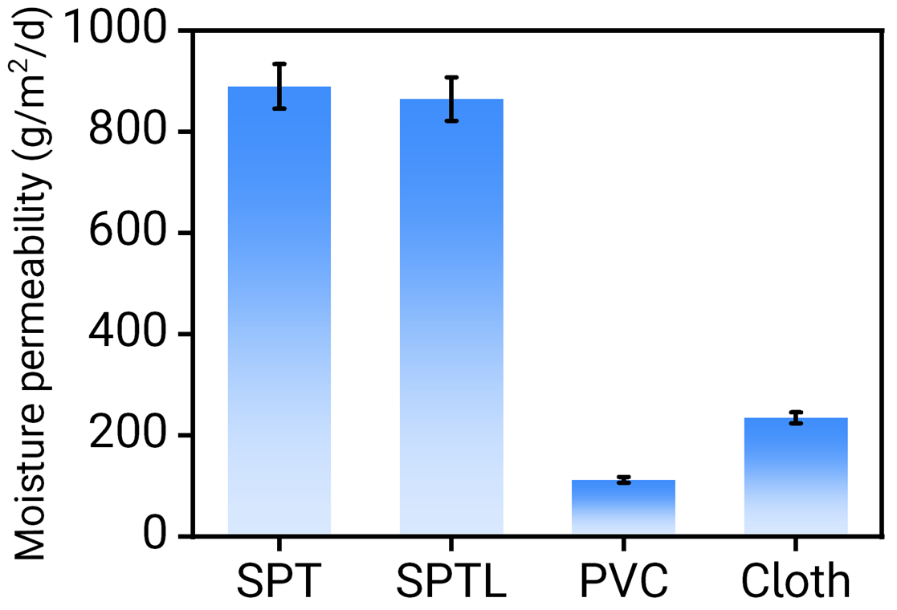


**Fig. S9 Moisture permeability comparison.** Water vapor permeability rate (WVTR) of SPT, SPTL, PVC tape, and cloth tape. (Error bars represent the SD, n = 3.)


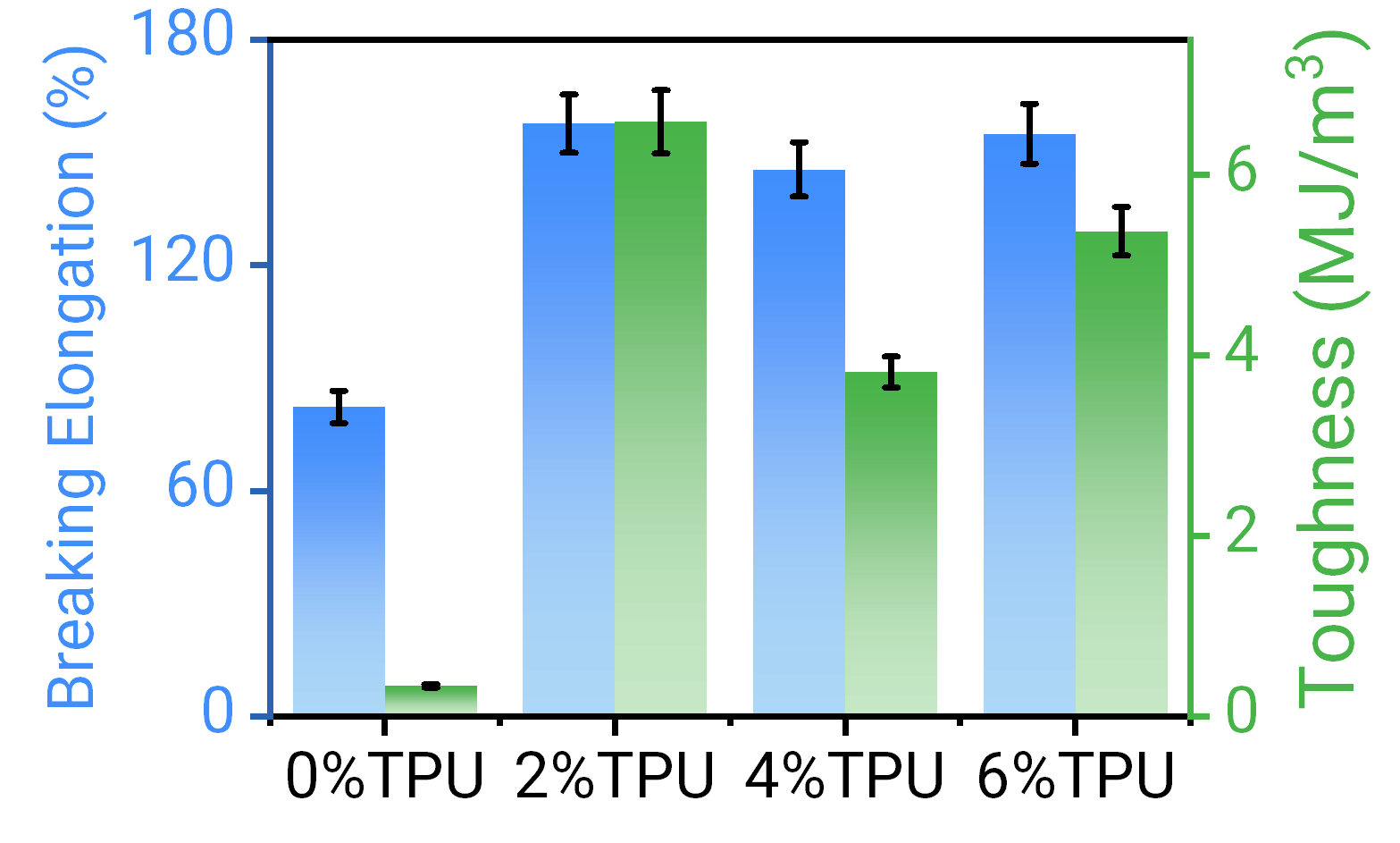


**Fig. S10 Mechanical optimization of PT nanofibers.** Bar chart illustrating the effect of different TPU contents on the breaking elongation and toughness of PT membranes (tensile speed: 10 mm/min). The comprehensive mechanical performance is optimal when the TPU content reaches 2%. (Error bars represent the SD, n = 3.)


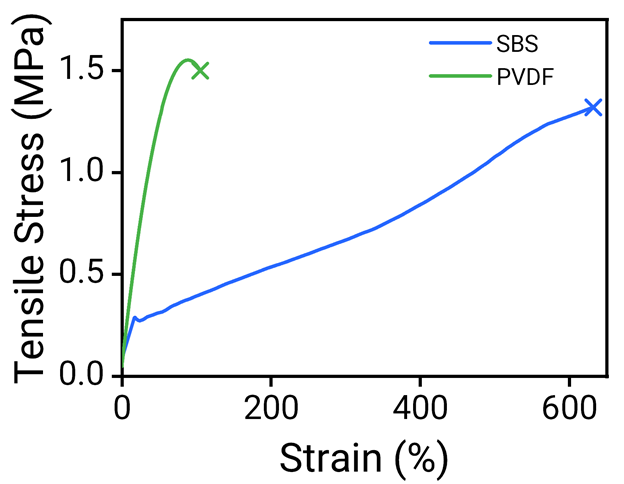


**Fig. S11 Mechanical performance comparison.** Stress-strain curves of electrospun SBS and PVDF membranes tested at a tensile speed of 10 mm/min.


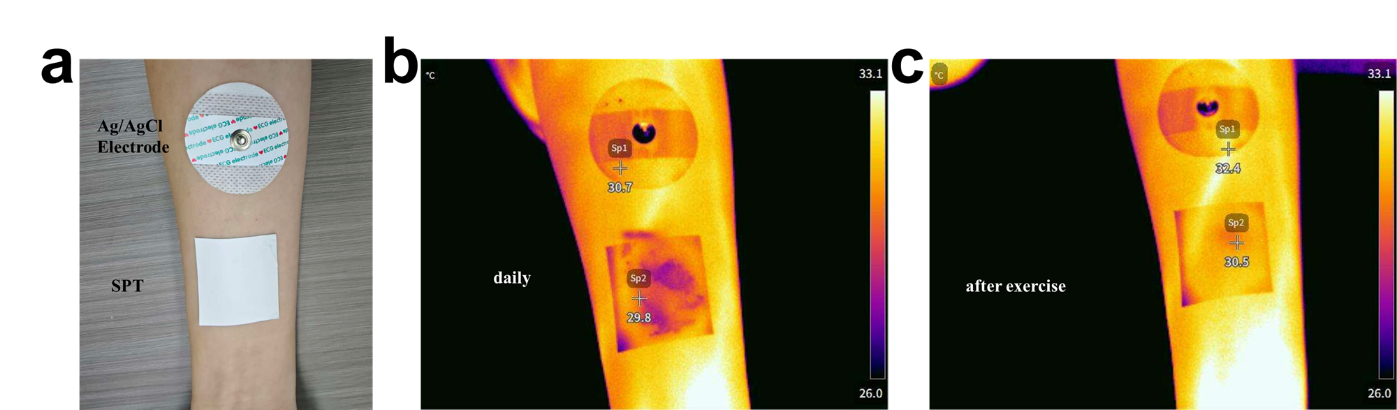


**Fig. S12 Thermal comfort and heat dissipation evaluation.** (a) Optical photographs of the SPT and commercial Ag/AgCl electrodes attached to the skin. (b, c) Infrared thermal images of the electrodes under daily wear conditions (b) and after exercise (c).


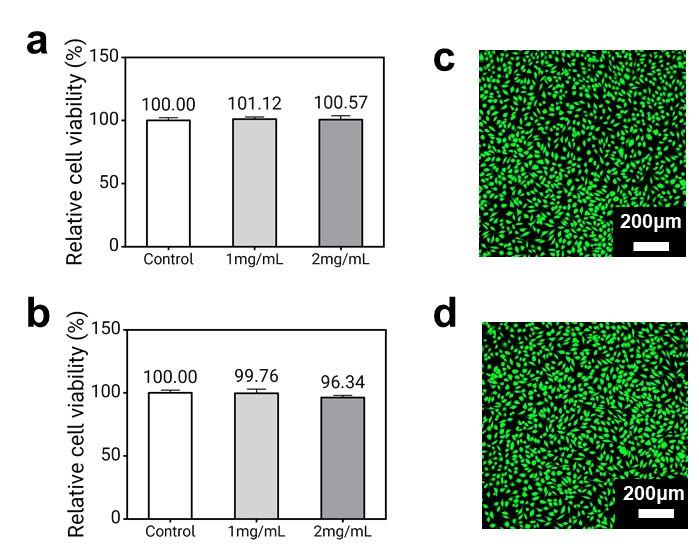


**Fig. S13 Biocompatibility evaluation of SPTL and SPT e-skins.** Cytotoxicity tests were conducted using electrospun extracts in accordance with the ISO 10993-5 standard. The extracts were prepared by soaking 9 mg/mL of electrospun materials in phosphate-buffered saline (PBS) at 37°C for 24 hours. (a, b) Relative cell viability of L929 cells treated with SPTL (a) and SPT (b) extracts diluted to 1 mg/mL and 2 mg/mL compared to the control group. (c, d) Live/dead staining confocal images of L929 cells incubated with SPTL extract (c) and SPT extract (d) (diluted to 1 mg/mL) for 24 hours (live cells: green; dead cells: red). (Data are presented as mean ± SD, n = 3.)


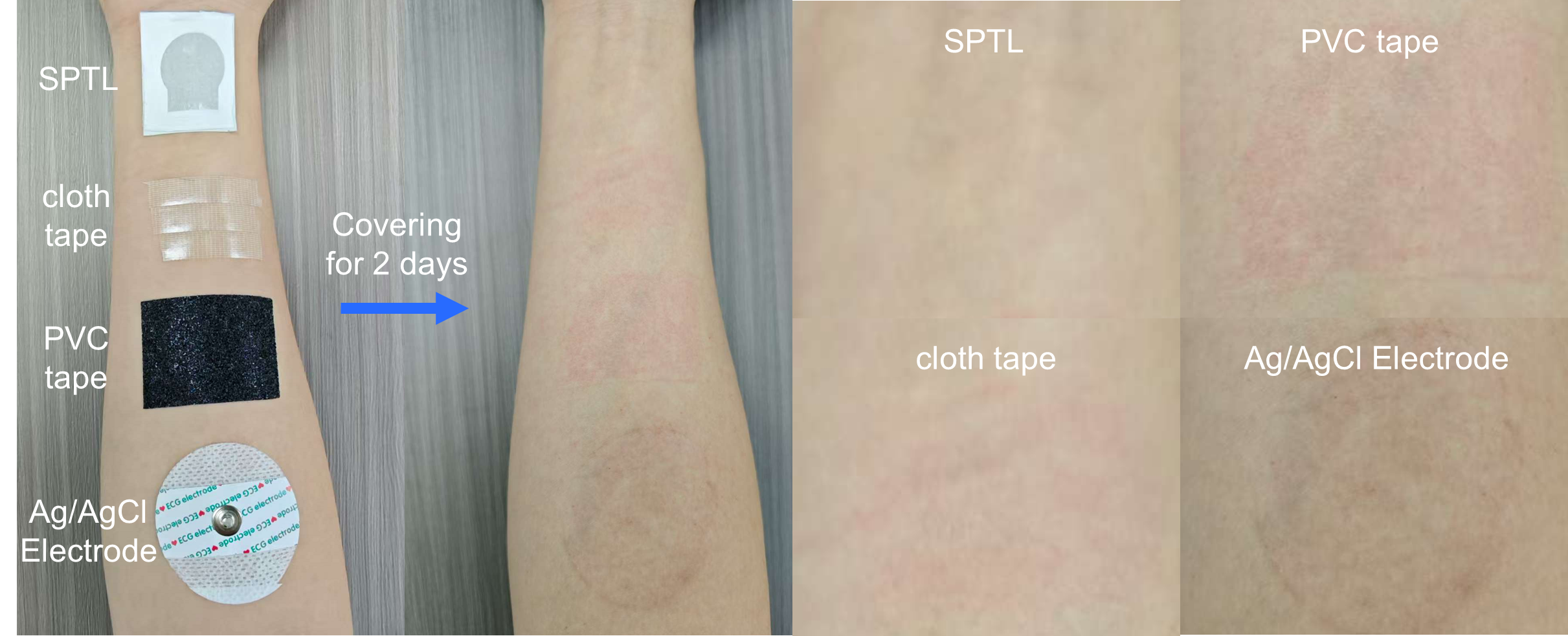


**Fig. S14 Skin irritation test on human skin.** Digital photographs showing the skin condition of a volunteer's right forearm before and after being covered with SPTL, cloth tape, PVC tape, and a commercial Ag/AgCl electrode for 2 days. The results highlight the skin-friendly nature of the SPTL e-skin compared to commercial adhesives.


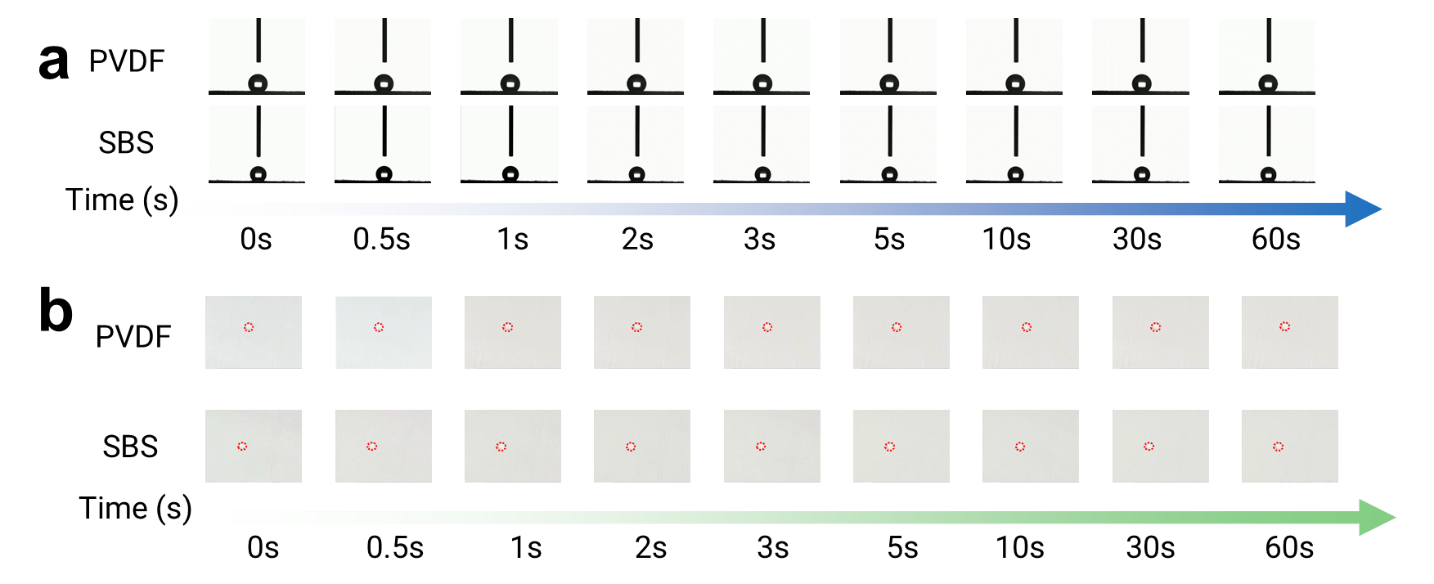


**Fig. S15 Dynamic wettability comparison.** (a) Time-dependent contact angle variation of a DI water droplet on PVDF and SBS surfaces. (b) Optical images showing the diffusion process of the water droplet on PVDF and SBS surfaces over 60 s.


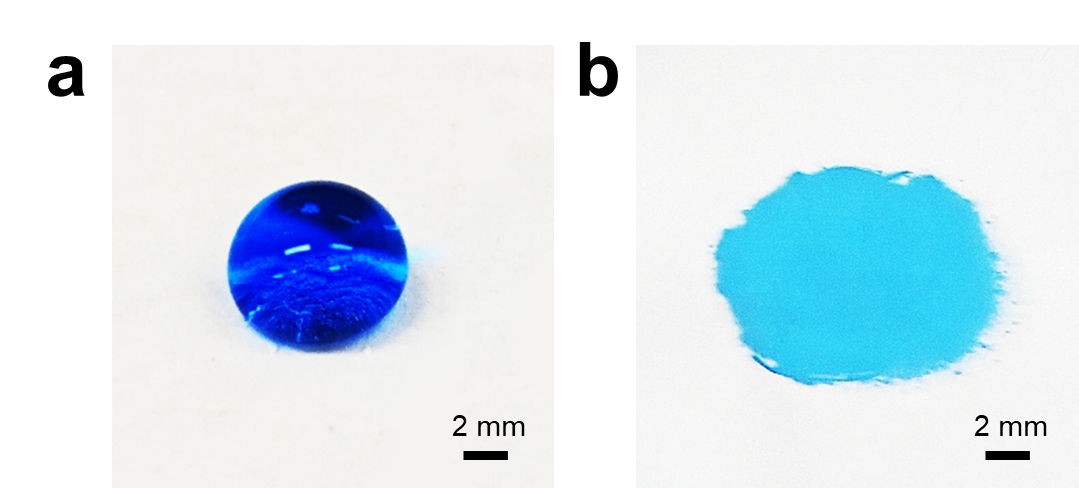


**Fig. S16 Macroscopic wettability demonstration.** Optical photographs showing the contact behavior of a DI water droplet (dyed with blue ink) on (a) SBS and (b) PT surfaces.


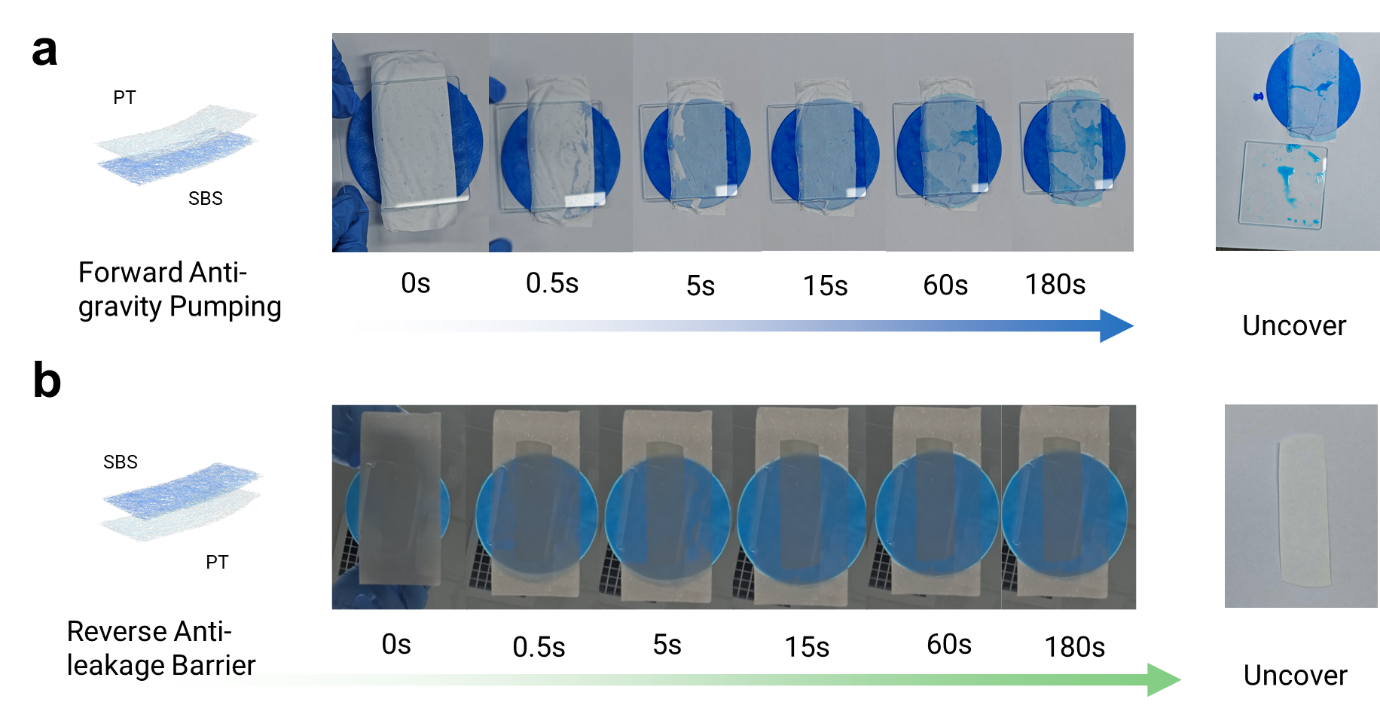


**Fig.S17 Macroscopic visual demonstration of the unidirectional liquid transport (liquid diode effect) under simulated wearing pressure.** (a) Forward anti-gravity pumping test: the membrane (SBS facing down, PT facing up) is placed on standard qualitative filter papers soaked with blue-dyed water, and a glass plate is manually applied. The top PT surface is rapidly and fully wetted within 15 s. The active upward water transport continues for 3 min, culminating in macroscopic water droplets remaining on the glass slide upon uncovering ("Uncover" panel), providing unequivocal evidence of transmembrane pumping. (b) Reverse anti-leakage barrier test: The membrane is sandwiched between a bottom pristine dry indicator filter paper and top blue-dyed wet filter papers. While the upper PT layer is rapidly wetted by the dyed water, the bottom indicator filter paper remains completely dry and white after the identical 3-min duration (shown in the "Uncover" panel). This physical evidence definitively confirms the strict prevention of liquid backflow.


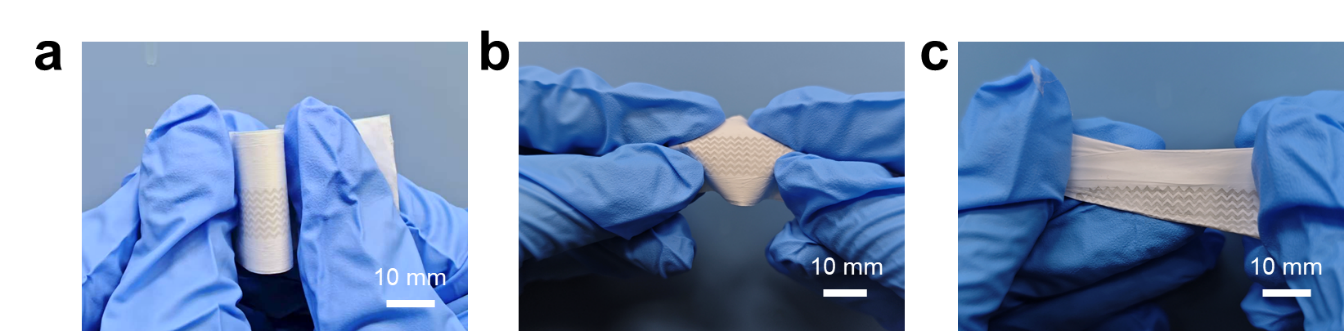


**Fig. S18 Mechanical flexibility and stability demonstration.** Optical photographs showing the flexible e-skin circuit under various deformations: (a) bending, (b) squeezing, and (c) twisting. These tests illustrate the mechanical stability of the circuit under physical stress.


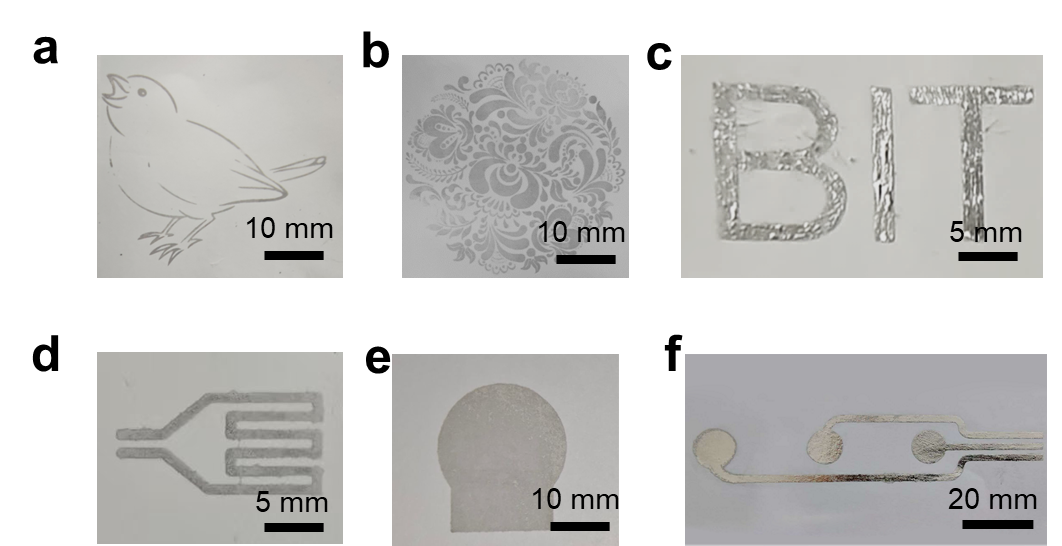


**Fig. S19 Screen-printing versatility and resolution.** (a–c) Various patterns screen-printed with LM ink, including a bird (a), a complex floral pattern (b), and the "BIT" logo (c). (d–f) Functional sensing circuits screen-printed with LM ink: (d) resistive e-skin (SPTL-3), (e) EMG and ECG electrodes (SPTL-4), and (f) EEG electrodes (SPTL-5).


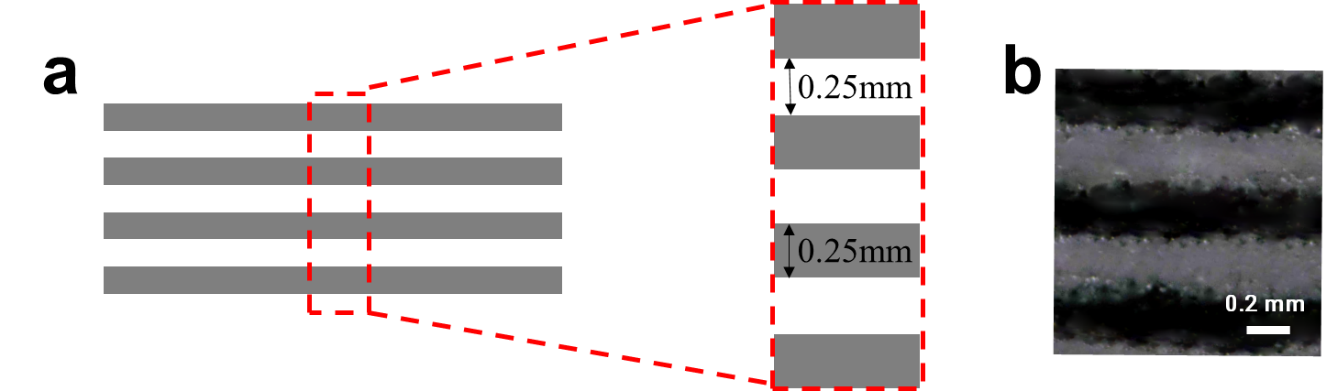


**Fig. S20 Printing resolution analysis.** (a) Schematic diagram and (b) optical microscopy image of the LM pattern printed on the SPT substrate, demonstrating a maximum achievable resolution of 250 μm.


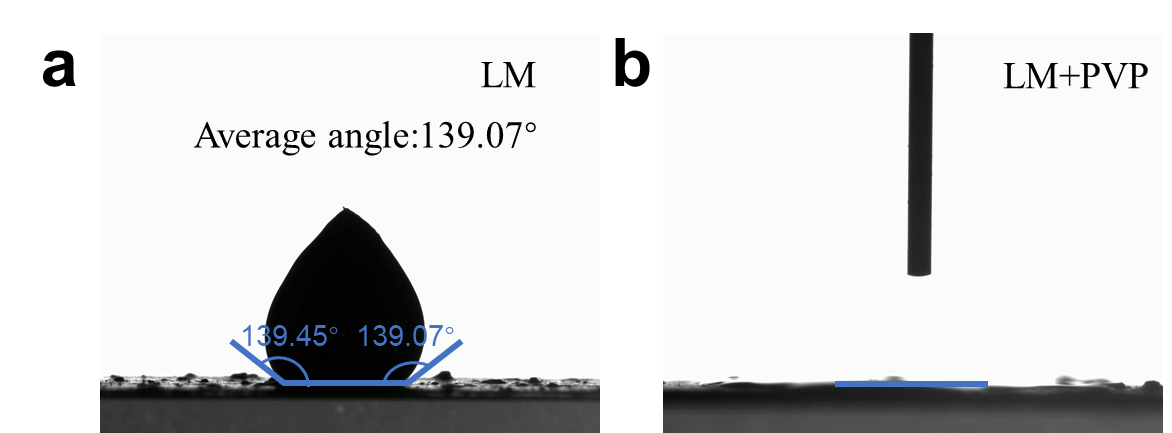


**Fig. S21 Static wettability comparison.** Optical images showing the contact angle of (a) a pure LM droplet (showing a high contact angle of ~139°) and (b) an LM/PVP ink droplet (showing significantly improved wettability) on the substrate.


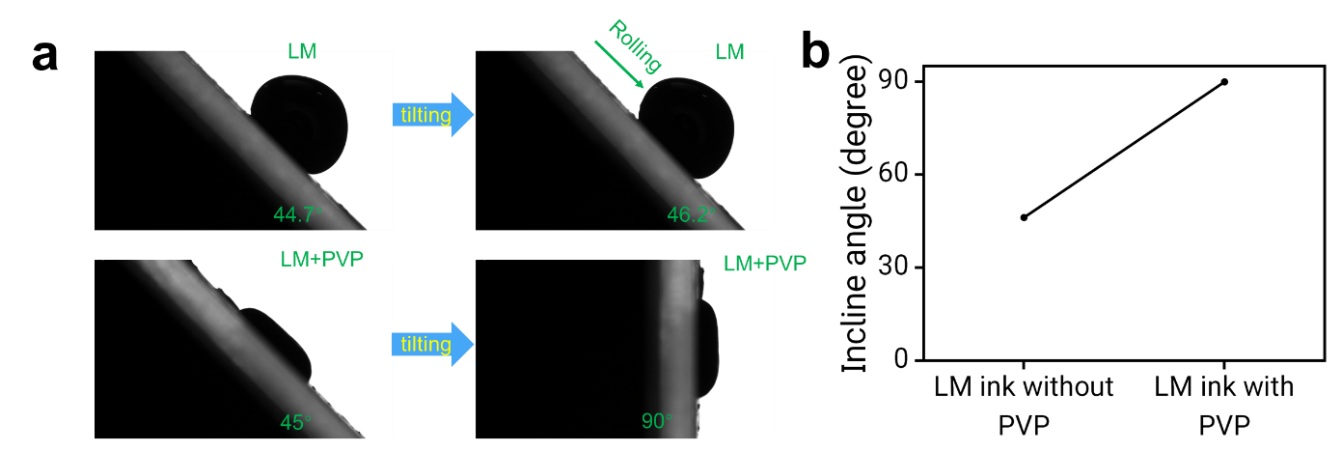


**Fig. S22 Adhesion performance evaluation.** (a) Digital photographs comparing adhesion on a tilted substrate: pure LM droplets (upper row) roll off easily, whereas LM/PVP ink droplets (lower row) remain adhered even at high tilt angles. (b) Comparison of the rolling angles (sliding angles) for LM droplets with and without PVP.


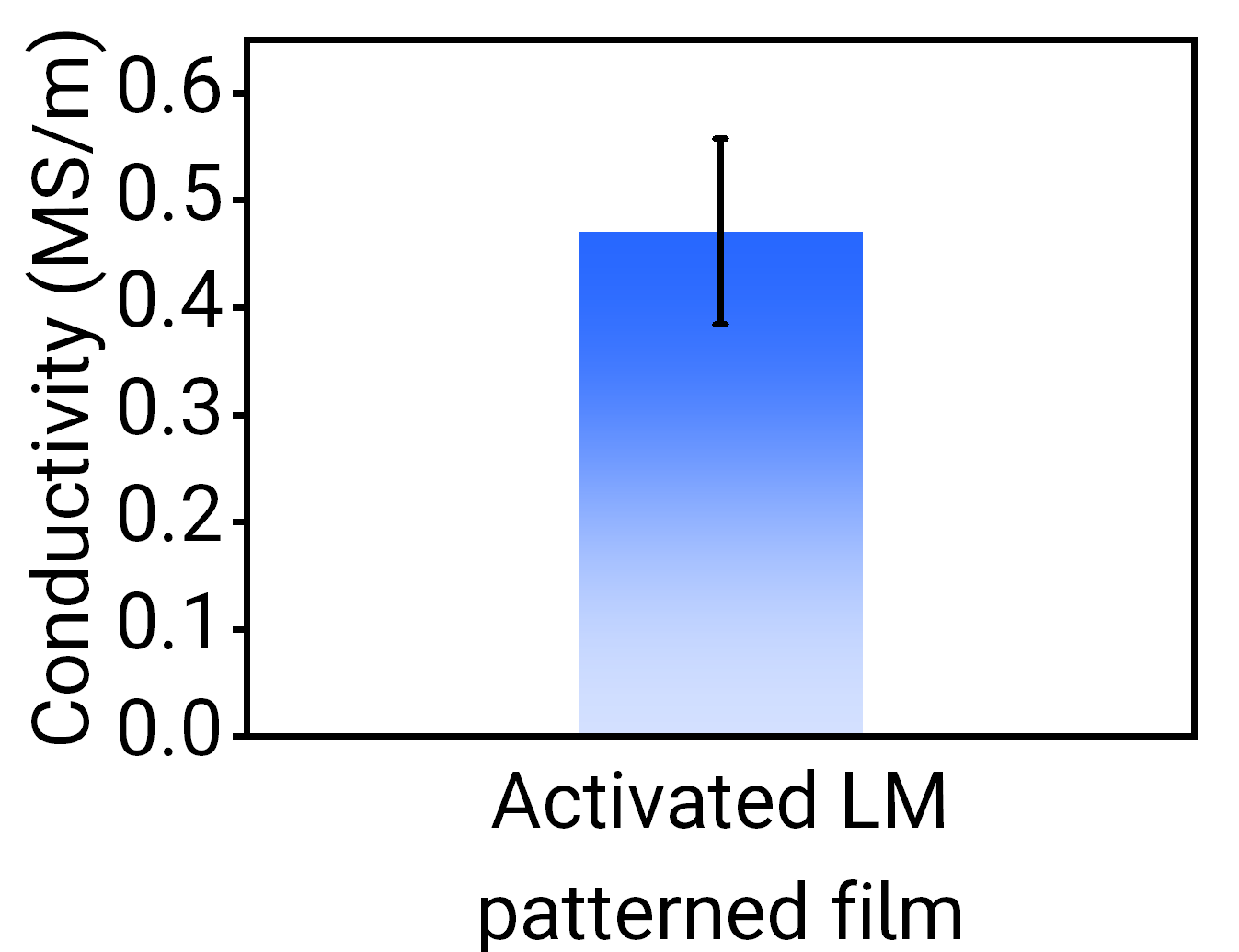


**Fig. S23 Electrical conductivity characterization.** Bar chart showing the electrical conductivity of the activated LM patterned film (0.47 MS/m). (Error bars represent the SD, n = 3.)


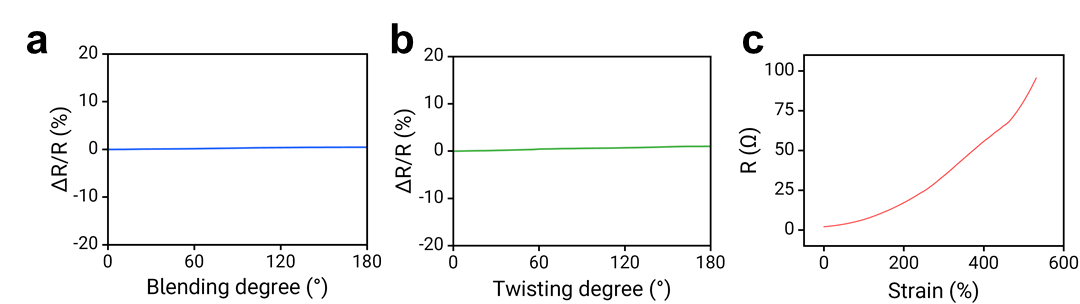


**Fig.** **S24 Mechanical robustness and electromechanical response.** Relative resistance change of the flexible resistive e-skin under (a) bending, (b) twisting, and (c) stretching. (Note: The e-skin shows stable resistance under bending and twisting, while exhibiting high sensitivity to stretching).


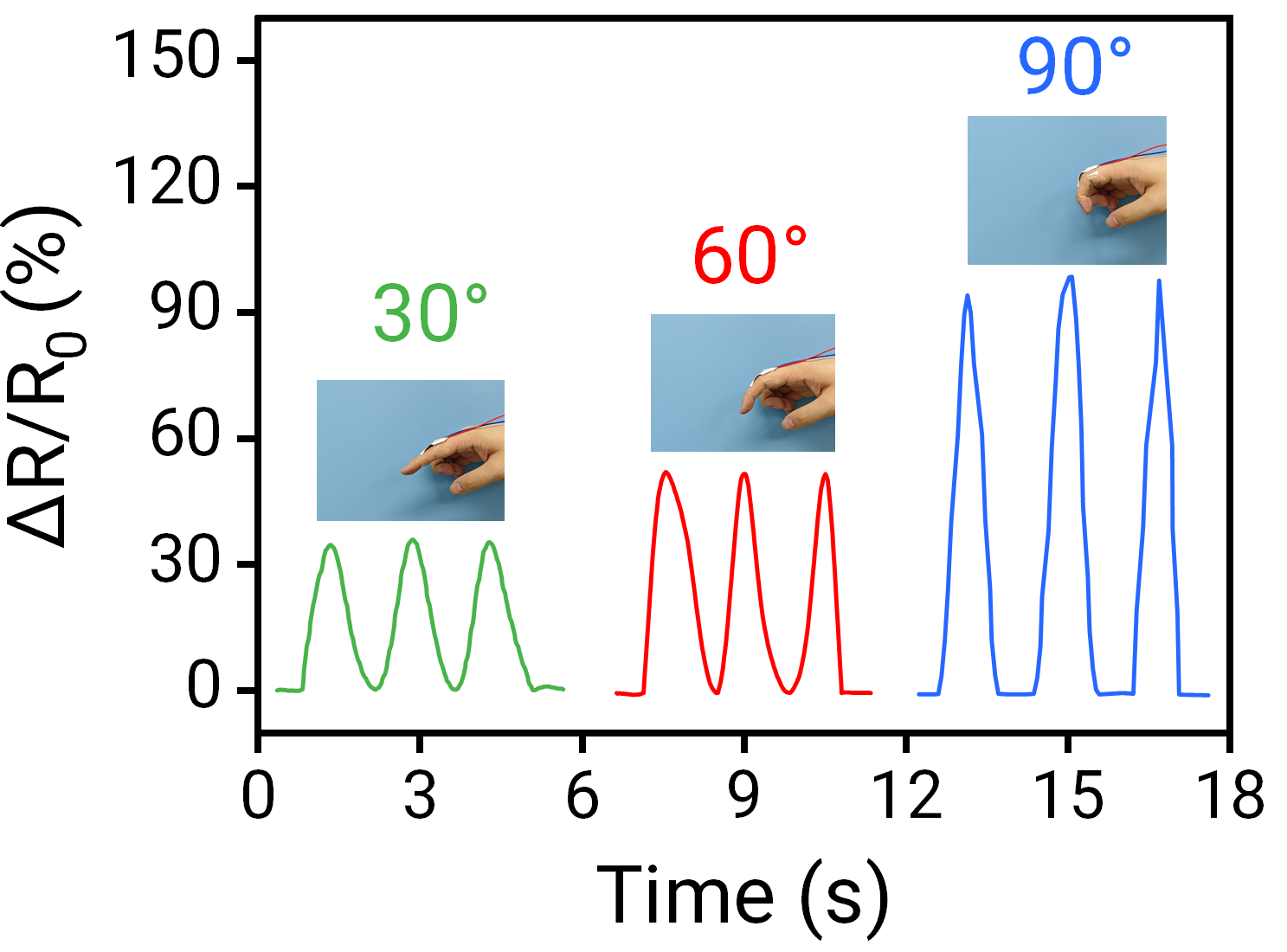


**Fig. S25 Real-time finger motion monitoring.** Relative resistance change of the resistive e-skin attached to a finger knuckle at different bending angles (30°, 60°, and 90°).


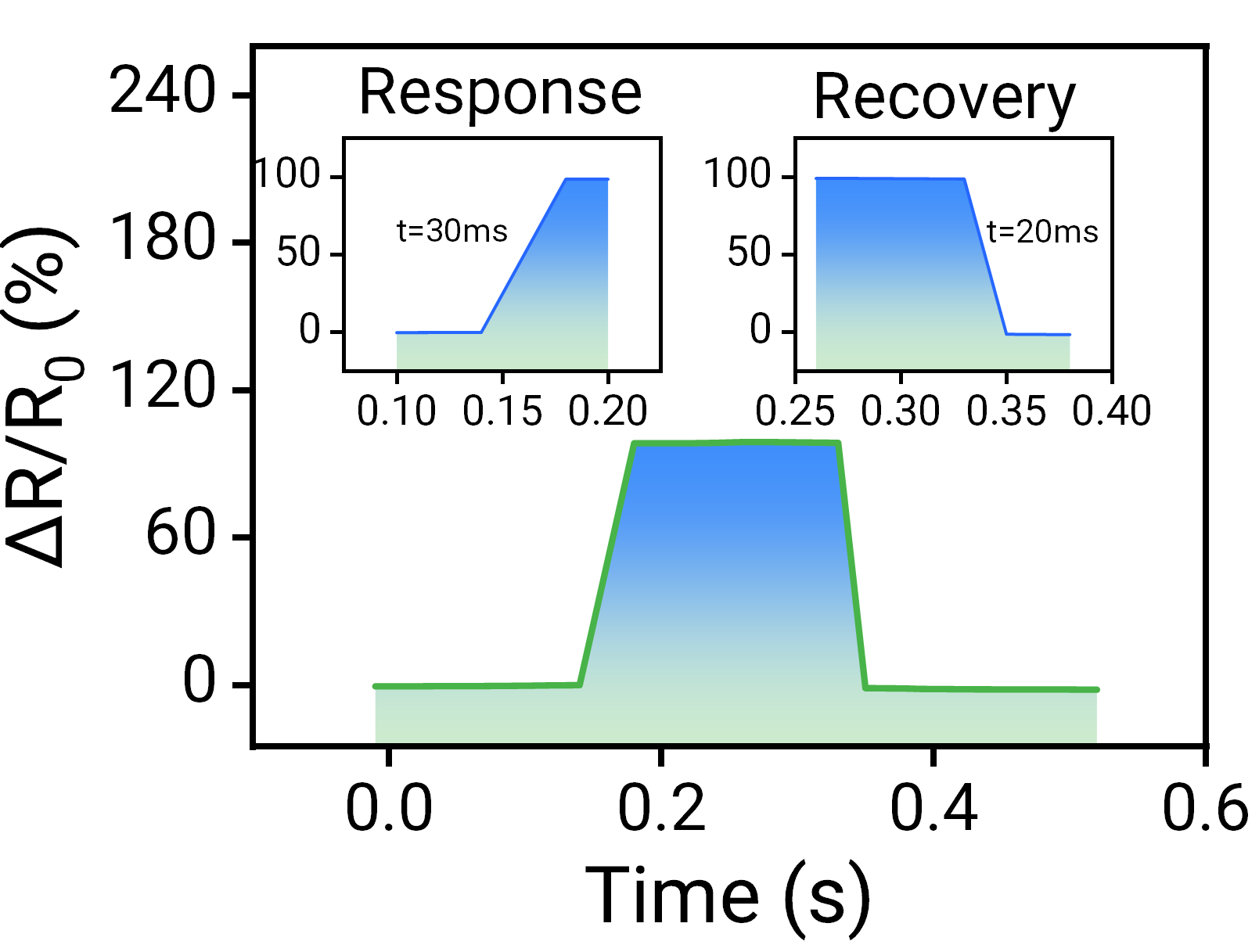


**Fig. S26 Dynamic response characteristics.** Response time (30 ms) and recovery time (20 ms) of the resistive e-skin attached to a finger knuckle during bending.


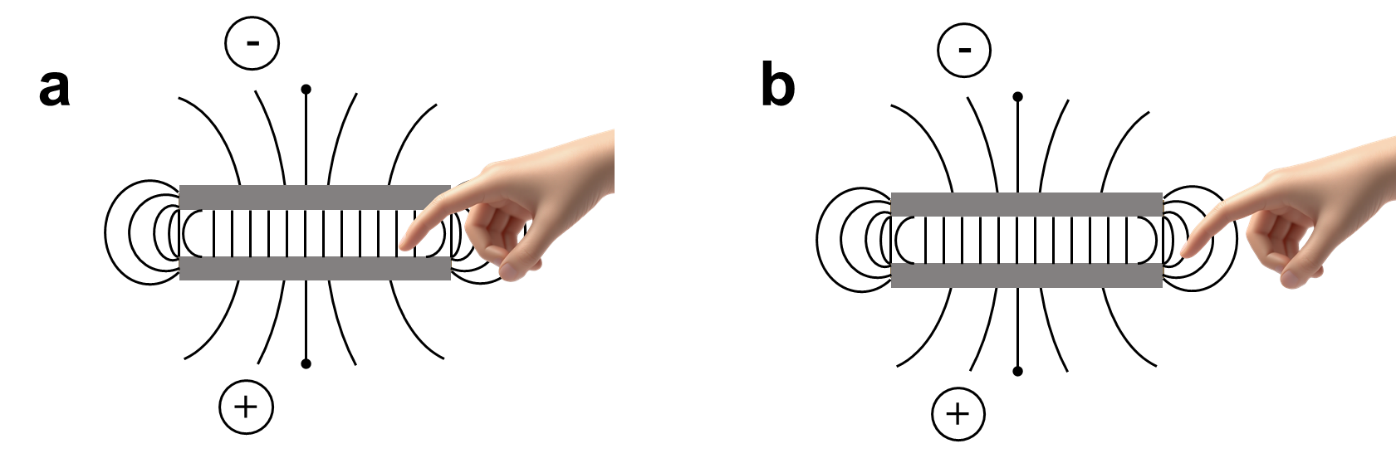


**Fig. S27 Sensing mechanism illustration.** Schematic diagrams illustrating the working principles and electric field distribution of the e-skin in (a) contact mode and (b) non-contact mode.


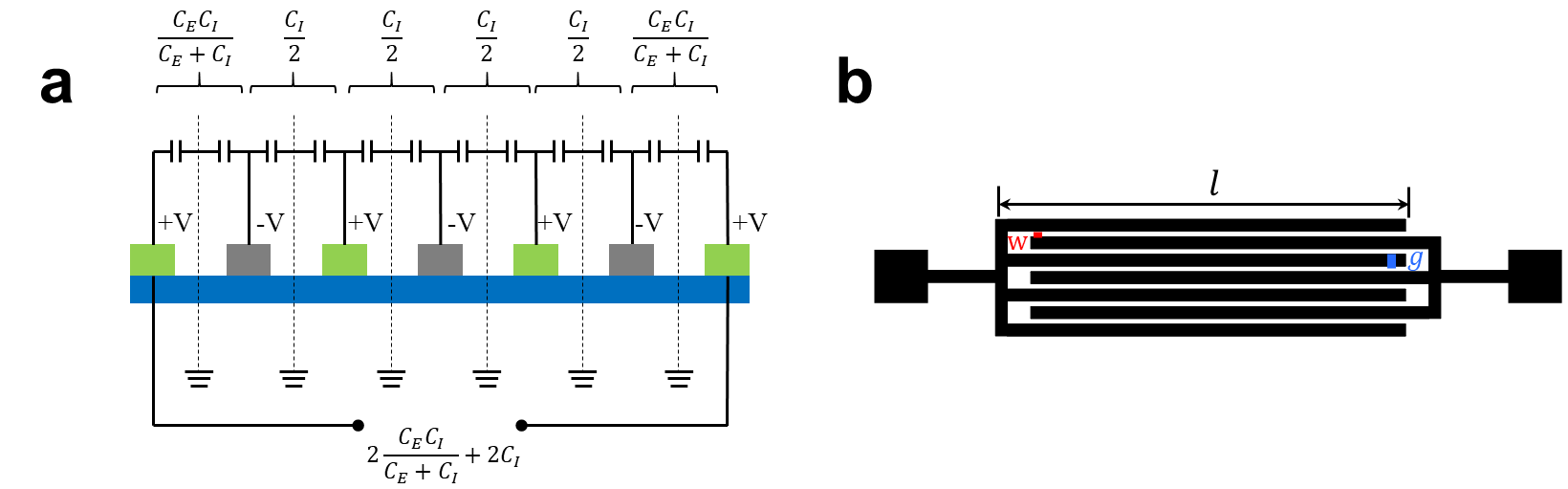


**Fig. S28 Theoretical modeling of the capacitive e-skin.** (a) Equivalent circuit model for calculating the capacitance of interdigital electrodes. (b) Schematic diagram showing the geometric parameters of the interdigital electrode structure.


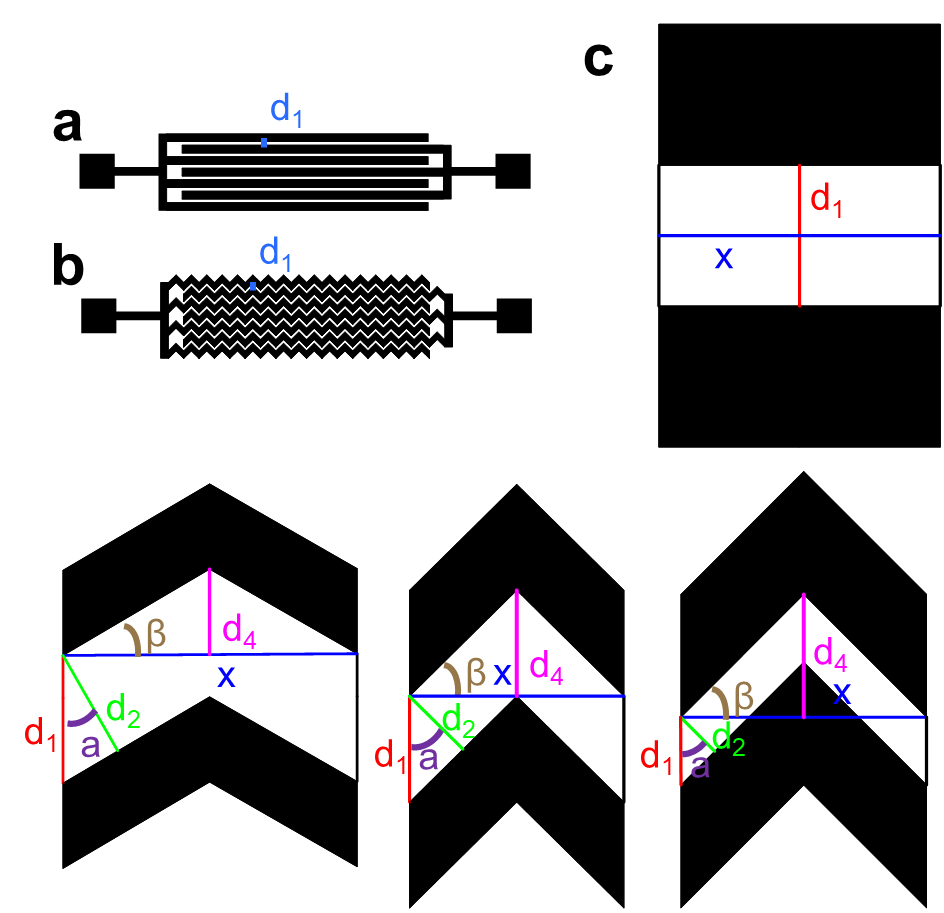


**Fig. S29 Geometric modeling of interdigital electrodes.** Schematic diagrams of (a) the straight circuit design and (b) the zigzag (wave-shaped) circuit design. (c) Detailed geometric parameters and angular analysis used to calculate the capacitance enhancement factor.

**Theoretical analysis:** Based on the geometric model presented in Fig. S28, we theoretically compared the capacitance of the e-skin with wave-structured electrodes (SPTL-1, denoted as $C_{1}$) with that of the e-skin with planar electrodes (SPTL-2, denoted as $C_{2}$). In this derivation, $d_{3}$ represents the electrode thickness, $x$ denotes the unit width, and $d_{1}$ indicates the horizontal spacing.

First, the capacitance of the planar structure ($C_{2}$) is calculated as follows:

$$C_{2}\text{=}\varepsilon_{0}\varepsilon_{r}\frac{S_{2}}{d_{1}}\text{=}\varepsilon_{0}\varepsilon_{r}\frac{xd_{3}}{d_{1}}$$

where $S_{2}= xd_{3}$ is the facing area of the planar electrode unit.

For the e-skin with wave-structured electrodes ($C_{1}$), the effective parameters are determined by the structural inclination angle $\alpha$ (which corresponds to $\beta$ in Fig. S28) and the wave height $d_{4}$. Additionally, let $d_{2}$ denote the effective perpendicular (normal) spacing between the inclined electrodes. The geometric relationships are:

$$d_{2}=d_{1}\cos\alpha$$

$$tan\alpha=\frac{2d_{4}}{x}$$

The effective facing area $S_{1}$ of the wave structure is derived by subtracting the non-overlapping region caused by the spacing $d_{1}$:

$$s_{1}=\frac{xd_{3}}{cos\alpha}-2d_{1}d_{3}\sin\alpha$$

Substituting $S_{1}$ and $d_{2}$ into the capacitance formula:

$$C_{1}\text{=}\text{ }\varepsilon_{0}\varepsilon_{r}\frac{S_{1}}{d_{2}}\text{=}\frac{\varepsilon_{0}\varepsilon_{r}(\frac{\text{x}}{cos\alpha}\text{-2}d_{1}\sin\alpha\text{）}d_{3}}{d_{1}\cos\alpha}$$

The ratio of $C_{1}$ to $C_{2}$ is then:

$$\frac{C_{1}}{C_{2}}=\frac{\frac{xd_{3}}{\cos^{2} \alpha d_{1}}-\frac{2d_{1}d_{3}\sin\alpha}{d_{1}\cos\alpha}}{\frac{xd_{3}}{d_{1}}}$$

$$\frac{C_{1}}{C_{2}}=\frac{1}{\cos^{2} \alpha}-\frac{2d_{1}\sin\alpha}{x\cos\alpha}=\sec^{2} \alpha-\frac{2d_{1}\tan\alpha}{x}$$

Using the identity $\sec^{2} \alpha= 1 + tan^{2}\alpha$ and substituting $\tan\alpha=\frac{2d_{4}}{x}$:

$$\frac{C_{1}}{C_{2}}=1+\tan^{2} \alpha-\frac{2d_{1}}{x}\tan\alpha$$

$$\frac{C_{1}}{C_{2}}=1+{(\frac{2d_{4}}{x})}^{2}-\frac{2d_{1}}{x}(\frac{2d_{4}}{x})$$

$$\frac{C_{1}}{C_{2}}=1+\frac{4d_{4}^{2}}{x^{2}}-\frac{4d_{1}d_{4}}{x^{2}}$$

$$\frac{C_{1}}{C_{2}}=1+\frac{4d_{4}}{x^{2}}(d_{4}-d_{1})$$

Thus, as long as the wave height $d_{4}$ is greater than the horizontal spacing $d_{1} \left( d_{4}> d_{1} \right)$, the ratio $\frac{C_{1}}{C_{2}}$ will be greater than 1, proving that the wave structure enhances capacitance.


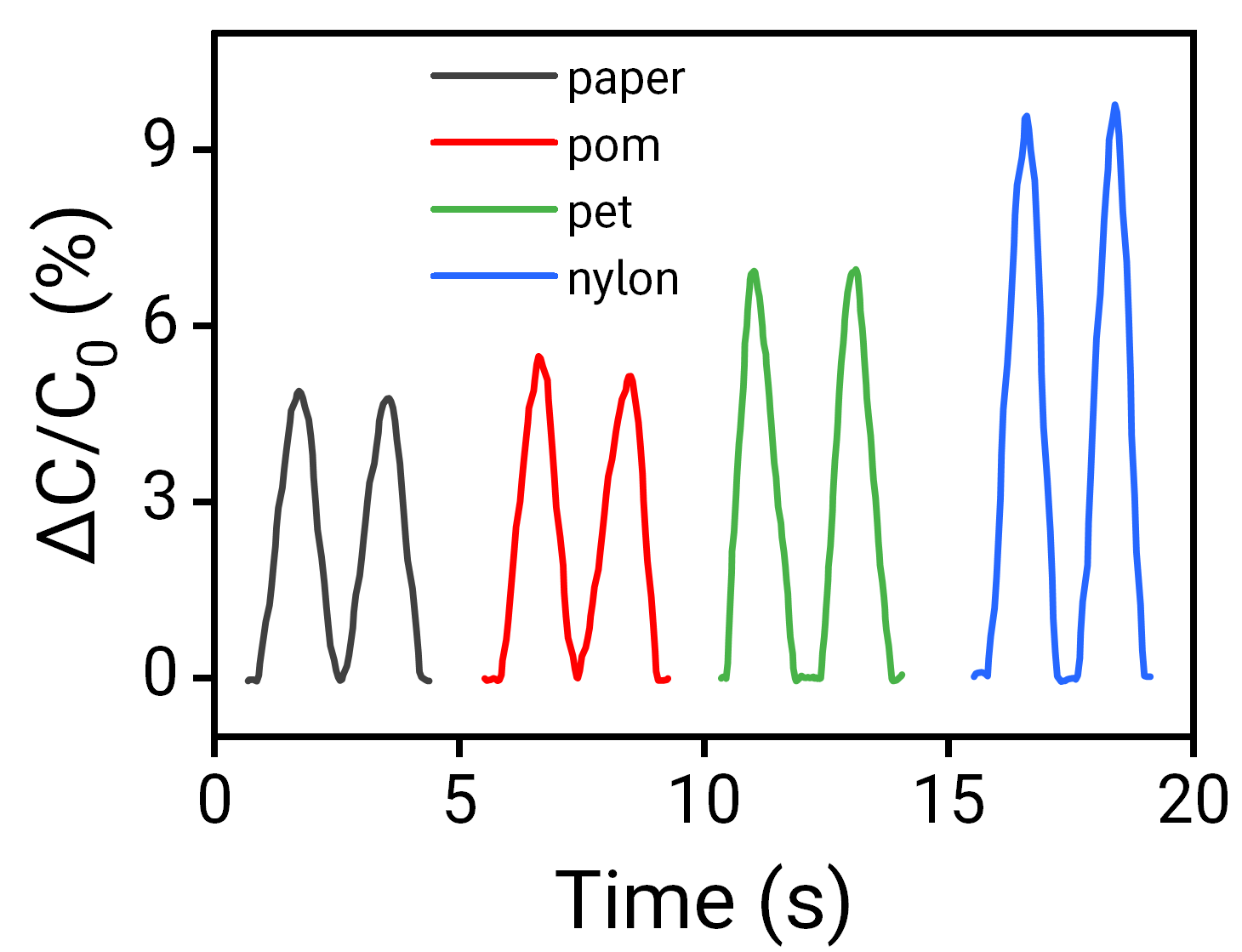


**Fig. S30 Material identification capabilities.** Relative capacitance change of the capacitive e-skin when contacting objects of different materials (paper, POM, PET, and nylon) with the same size.


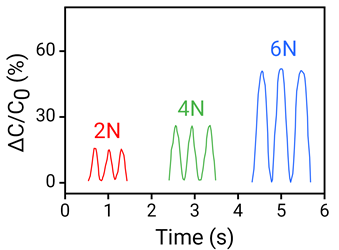


**Fig. S31 Pressure discrimination capabilities.** Relative capacitance change of the capacitive e-skin under different applied pressures (2 N, 4 N, and 6 N).

**Fig. S32 Frequency response characteristics.** Comparison diagram of the differences in relative capacitance change under different pressing frequencies.


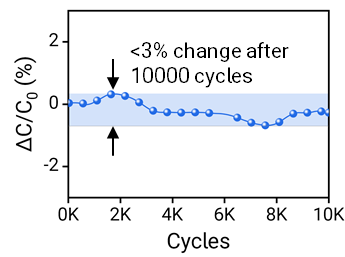


**Fig. S33 Long-term mechanical durability.** Relative capacitance change of the capacitive e-skin after 10,000 cycles of pressing.


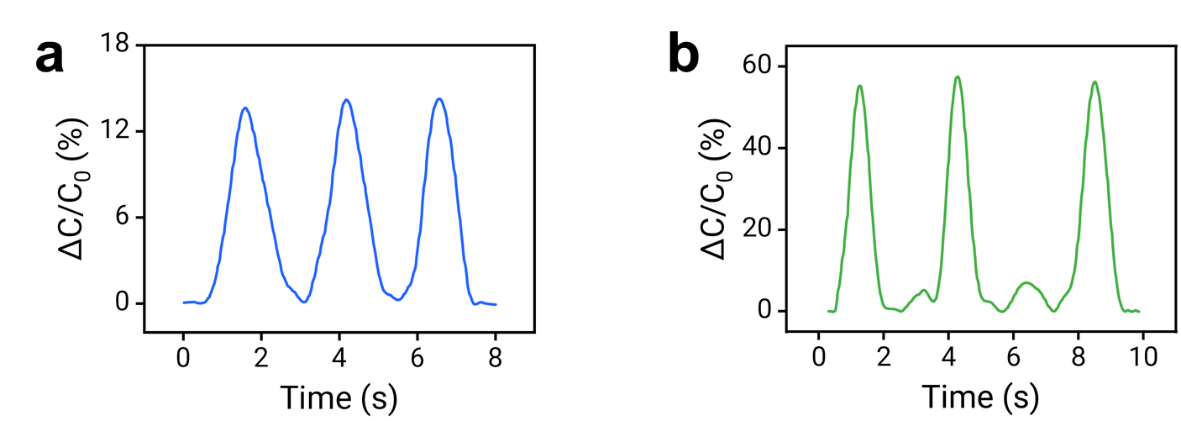


a

b

**Fig. S34 Relative capacitance response of the e-skin under micro-deformation.** (a) Relative capacitance change when a 0.15 g lightweight object is placed on the e-skin. (b) Relative capacitance change of the e-skin attached to the larynx during water swallowing.


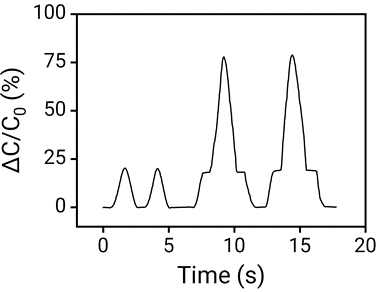


**Fig. S35 Real-time relative capacitance changes of the SPTL-1 e-skin during a continuous approaching, holding, and pressing sequence.** The distinct waveform profiles and amplitude thresholds (including the non-contact precursor plateau and the sharp contact peak) enable reliable differentiation between non-contact and contact sensing modes.


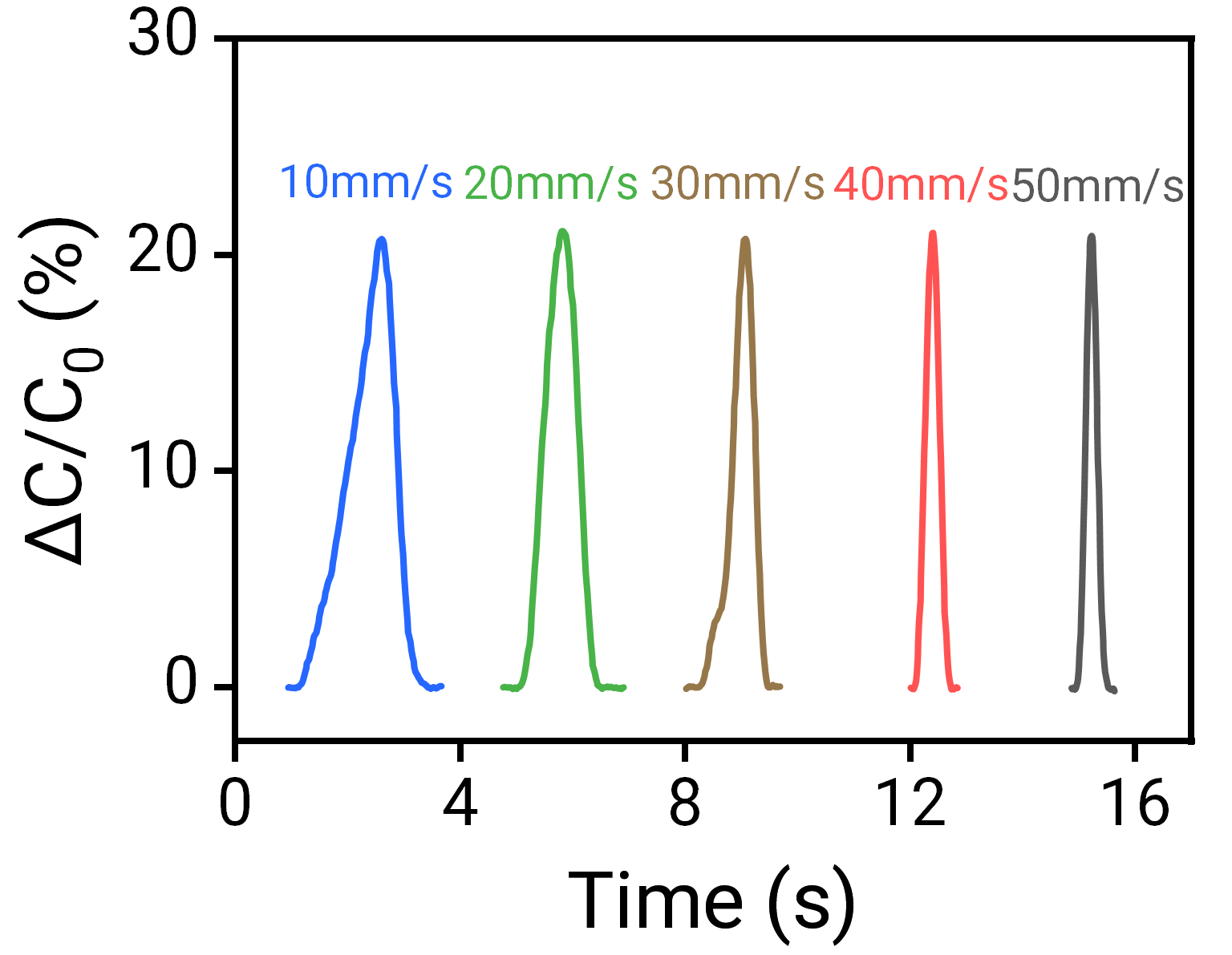


**Fig. S36 Influence of approach speed on non-contact sensing signals.** Relative capacitance change when a finger approaches the e-skin at different speeds (10 mm/s to 50 mm/s) in non-contact mode.


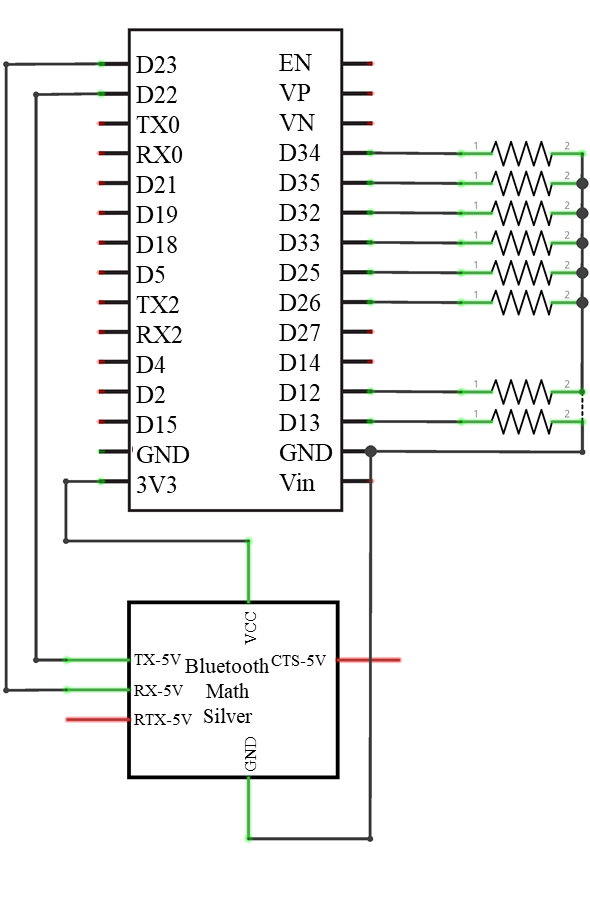


**Fig. S37 Schematic diagram of information receiving and servo drive control for the quadruped robot based on EMG signals.** After the EMG signals are sent as control commands, they are received by the HC-06 Bluetooth module and transmitted to the ESP32 microcontroller, which regulates the servo operating states according to the commands.


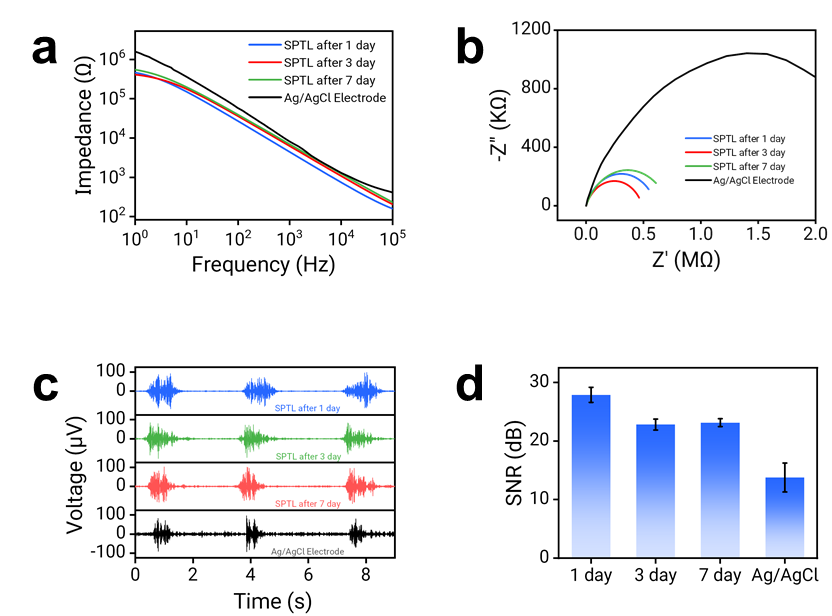


**Fig. S38 Long-term stability and aging tests of the SPTL e-skin.** (a) Bode plots, (b) Impedance plots, (c) EMG signal plots, and (d) SNR of the SPTL e-skin (after scraping off the surface polymer material and stored for 1, 3, and 7 days) and Ag/AgCl electrodes. (Error bars represent the SD, n = 3.)


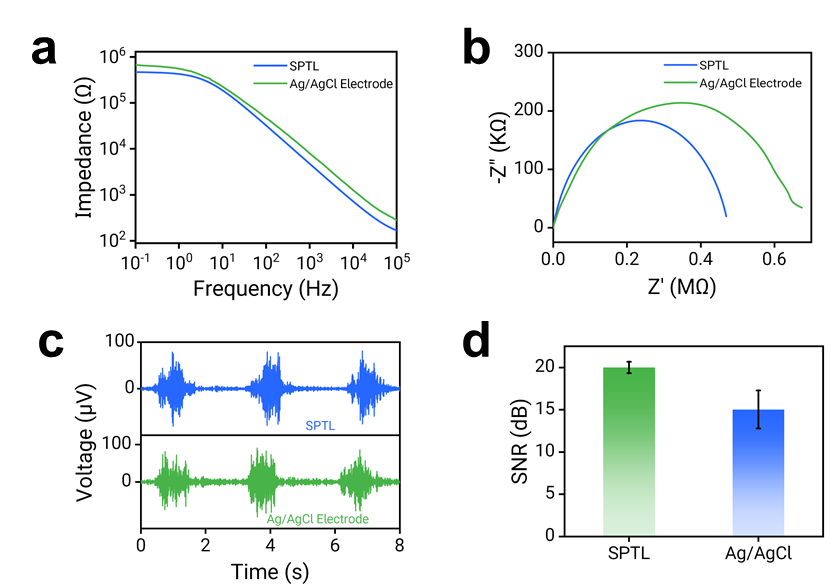


**Fig. S39 Signal performance stability under simulated sweating conditions.** (a) Bode plots, (b) Impedance plots, (c) EMG signal plots, and (d) SNR of SPTL-4 and Ag/AgCl electrodes measured after dropping 3 mL of artificial sweat on the skin side to simulate heavy exercise. (Error bars represent the SD, n = 3.)


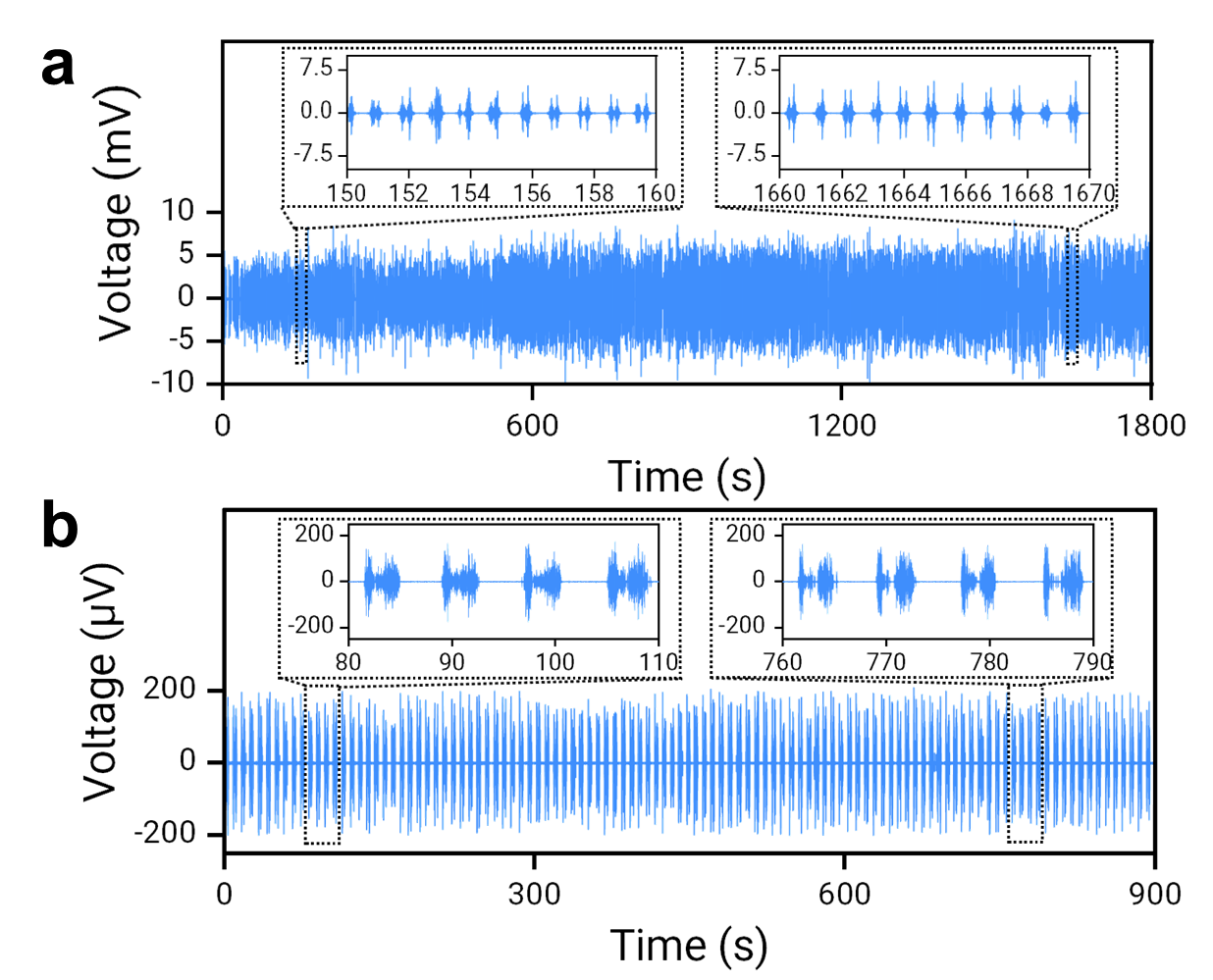


**Fig. S40 Real-time EMG (electromyogram) monitoring during prolonged dynamic exercises.** (a) A 30-minute intensive spinning bike exercise. The main plot displays the continuous macroscopic recording over the entire 1800 s period. Two insets present randomly selected 10-s intervals (150–160 s and 1660–1670 s) extracted from the early and late stages. (b) A 15-minute dynamic dumbbell lifting exercise. The plot displays the continuous recording over the 900 s period, with two insets presenting 30-s intervals (80–110 s and 760–790 s) extracted from the early and late stages. The results demonstrate that the SPTL-4 e-skin maintains high-fidelity signal acquisition and operational robustness despite profuse natural sweating and continuous dynamic movement.


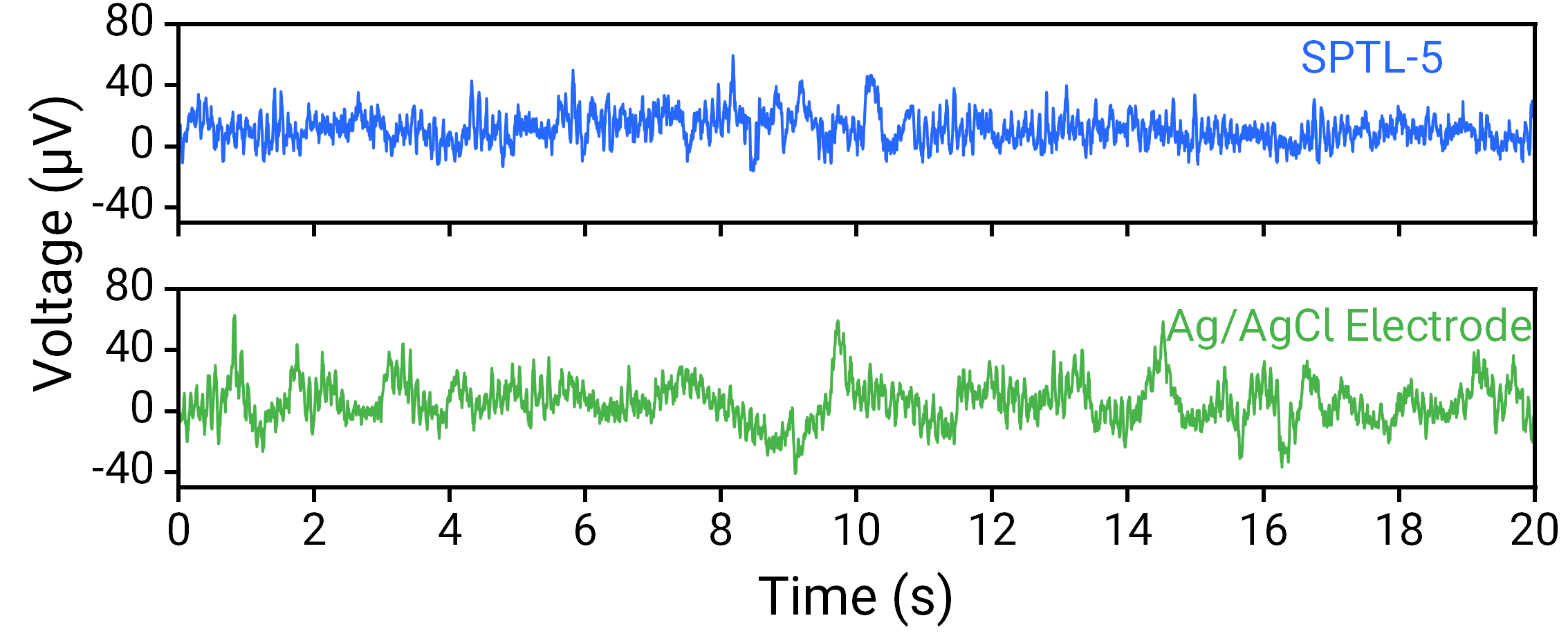


**Fig. S41 EEG signal monitoring capability.** Comparison of resting-state EEG signals recorded by SPTL and Ag/AgCl electrodes, showing characteristic alpha rhythms at approximately 10 Hz.


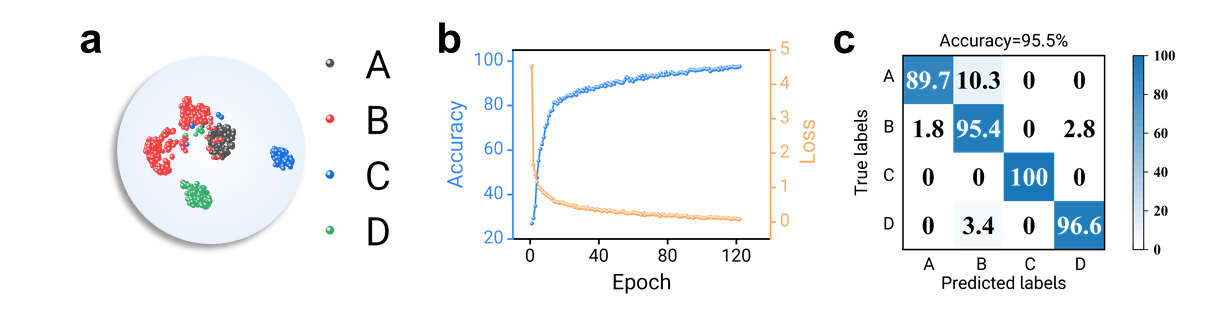


**Fig. S42 Machine learning evaluation for handwritten letter recognition.** (a) t-SNE projection of high-dimensional latent features of handwritten letters A, B, C, and D using transfer learning. (b) Learning accuracy and loss curves over 120 epochs. (c) Confusion matrix of the four different handwritten letters.

**Table S1** Performance comparison of the SPTL e-skin with other reported flexible devices

| **Reference** | Sensitivity(kPa^-1^) | Durability(Cycles) | Response Time (ms) | Non-contact Mode | Contact Mode | Strain Range (%) |
| --- | --- | --- | --- | --- | --- | --- |
| This Work | 7.39 | 10000 | 20 | YES | YES | 627 |
| PANI/MCCTO/PEO [S1] | 0.12 | N/A | 2100 | NO | YES | N/A |
| Ni/PI [S2] | 0.004 | 1000 | 40 | NO | YES | N/A |
| LIG/PVP [S3] | 0.338 | 3000 | 31 | NO | YES | 25±1.5 |
| LM/TPU [S4] | 0.055 | 6000 | 50 | NO | YES | 500 |
| TPU/LM [S5] | 0.324 | N/A | 161.5 | YES | YES | 190±5 |
| APPS [S6] | 0.54 | N/A | 12 | NO | YES | N/A |
| CNT/Mxene/PDMS [S7] | 0.091 | 6000 | 71 | NO | YES | N/A |
| TCE [S8] | 2.43 | 2600 | N/A | NO | YES | 673 |

N/A means “not reported”

**Supplementary References**

1. B. Dhamodharan, S. Rajiv et al., Flexible, hybrid nanofibrous capacitive pressure sensor for self-healing electronics. New J. Chem. **49**, 15343–15359 (2025). <https://doi.org/10.1039/d5nj02388b>
2. L. Xia, W. Xiao, L. Li, X. Liu, Q. Zhuang et al., High-Performance Flexible Capacitive Pressure Sensor Based on a Spiked Nickel/Polyimide Composite Nanofiber Membrane. ACS Sens. **10**, 1450–1460 (2025). <https://doi.org/10.1021/acssensors.4c03495>
3. Z. Jin, S. Dong, X. Liu, X. Zheng, J. Guo et al., Hierarchical microstructure-enhanced flexible capacitive pressure sensor for high-accuracy fruit identification. Chem. Eng. J. **520**, 166137 (2025). <https://doi.org/10.1016/j.cej.2025.166137>
4. Y. Chen, T. Feng, M. Peng, F. Qin et al., Ultra-high Filling Ratio of Non-Percolative Rapeseed-Shaped Liquid Metal Fiber Mats for Pressure Sensors Via Electrospinning Aided Inhomogeneous Dispersion. Adv. Fiber Mater. **7**, 633–644 (2025). <https://doi.org/10.1007/s42765-025-00515-y>
5. H.-C. Xu, Y. Liu, Y.-P. Mo, Z.-Y. Chen, X.-J. Pan et al., All-fiber anti-jamming capacitive pressure sensors based on liquid metals. Rare Met. **44**, 4839–4850 (2025). <https://doi.org/10.1007/s12598-024-03071-3>
6. X. Wang, Y. Li, Y. Wang, W. Huang, X. Zhao et al., Fabrication method and various application scenarios of flexible capacitive pressure sensor based on direct formation of conical structure. Chem. Eng. J. **496**, 153957 (2024). <https://doi.org/10.1016/j.cej.2024.153957>
7. Y. Chen, T. Feng, C. Li, F. Qin et al., Comprehensive and Robust Anti-Jamming Dual-Electrode Pair Sensor. Small **20**, 2406739 (2024). <https://doi.org/10.1002/smll.202406739>
8. P. Guruprasad Reddy, A. Barua, T. Laukkanen, B. Mostafiz, T. Tirri et al., Sustainable cross-linked poly(glycerol–co–δ–valerolactone) urethane substrates and multipurpose transparent electrodes for wearable electronics. Chem. Eng. J. **495**, 153531 (2024). <https://doi.org/10.1016/j.cej.2024.153531>
